# Organic room-temperature phosphorescence from halogen-bonded organic frameworks: hidden electronic effects in rigidified chromophores†

**DOI:** 10.1039/d0sc04646a

**Published:** 2020-11-05

**Authors:** Jiawang Zhou, Ljiljana Stojanović, Andrey A. Berezin, Tommaso Battisti, Abigail Gill, Benson M. Kariuki, Davide Bonifazi, Rachel Crespo-Otero, Michael R. Wasielewski, Yi-Lin Wu

**Affiliations:** Department of Chemistry, Institute for Sustainability and Energy at Northwestern, Northwestern University Evanston Illinois 60208-3113 USA m-wasielewski@northwestern.edu; School of Biological and Chemical Sciences, Queen Mary University of London London E1 4NS UK r.crespo-otero@qmul.ac.uk; School of Chemistry, Cardiff University Cardiff CF10 3AT UK WuYL@cardiff.ac.uk; Institute of Organic Chemistry, Faculty of Chemistry, University of Vienna Währinger Str. 38 Vienna 1090 Austria

## Abstract

Development of purely organic materials displaying room-temperature phosphorescence (RTP) will expand the toolbox of inorganic phosphors for imaging, sensing or display applications. While molecular solids were found to suppress non-radiative energy dissipation and make the RTP process kinetically favourable, such an effect should be enhanced by the presence of multivalent directional non-covalent interactions. Here we report phosphorescence of a series of fast triplet-forming tetraethyl naphthalene-1,4,5,8-tetracarboxylates. Various numbers of bromo substituents were introduced to modulate intermolecular halogen-bonding interactions. Bright RTP with quantum yields up to 20% was observed when the molecule is surrounded by a Br⋯O halogen-bonded network. Spectroscopic and computational analyses revealed that judicious heavy-atom positioning suppresses non-radiative relaxation and enhances intersystem crossing at the same time. The latter effect was found to be facilitated by the orbital angular momentum change, in addition to the conventional heavy-atom effect. Our results suggest the potential of multivalent non-covalent interactions for excited-state conformation and electronic control.

## Abstract 2

The number and position of halogen substituents in purely organic π–π* chromophores critically affect the efficiency of phosphorescence.

## Graphical abstract

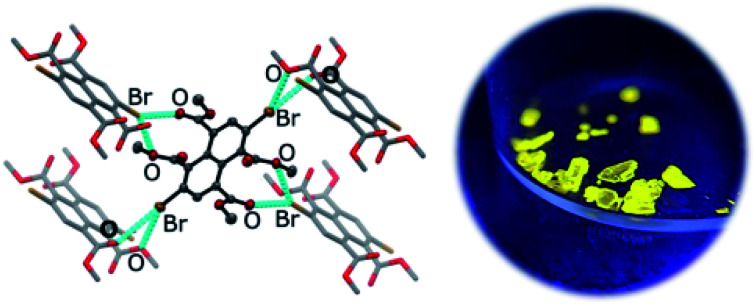

## Introduction

Room temperature phosphorescence (RTP) has received increasing interest due to the potential it presents for photonic devices, bio-imaging, anti-counterfeiting, and night-vision applications.^1–3^ Until recent years, the main sources of RTP luminophores have been inorganic or organometallic complexes, due to the presence of metal atoms being able to promote singlet-to-triplet intersystem crossing (ISC) in the excited states. However, heavy metal complexes or inorganic materials can often be toxic and expensive; through the study of purely organic phosphors, the applications of phosphorescence materials can expand by becoming more biocompatible, cheaper to acquire, and environmentally safer.^4,5^ While there are many benefits of organic phosphors compared to those containing heavy metals, achieving RTP from purely organic molecules has proven a challenge on account of slow ISC rates and competitive non-radiative processes, in particular.

In recent decades, organic phosphorescence has become a more widely explored topic due to the discovery of long-lasting RTP by utilising crystallisation,^6–8^ aggregation,^9,10^ halogen bonding,^11–14^ heavy atoms,^15^ and carbonyl substituents^16–18^ to circumvent the aforementioned issues.^19–28^ Although spin–orbit coupling (SOC) in organic molecules is usually small (on the order of 1 cm^−1^, *cf.* 10^2^ to 10^3^ cm^−1^ for organometallic complexes), the introduction of a carbonyl functionality to aromatic rings often opens up a ^1^(n–π*) → ^3^(π–π*) (or ^1^(π–π*) → ^3^(n–π*)) channel with SOC ∼100 cm^−1^.^29–33^ Such a small increase is sufficient to allow efficient ISC and populate the triplet of, for instance, benzophenone or benzaldehyde with a near-unitary quantum efficiency.^34,35^ The structure of the as-generated triplet states can be rigidified in the solid state with the aid of non-covalent interactions (*e.g.* hydrogen and halogen bonds)^11–13,20^ to suppress non-radiative vibrational relaxation, resulting in nearly quantitative RTP quantum yields in the solid state.^19,36^

Combining these design principles, the Kim group reported seminal work on efficient RTP luminophores based on 2,5-bis(hexyloxy)-4-bromobenzaldehyde.^37^ The linear C=O⋯Br halogen-bonding interactions^38,39^ present in the solid state were suggested to be the major reason to avoid energy dissipation through vibrational motions. The proximity of a fourth-row Br element to the C=O group, where the non-bonding electrons originate in the n–π* transition, is believed to enhance SOC as well.^40,41^ In fact, in a later study by Kim and Dunietz, it was found that moving the Br substituent from the *para* to the *ortho* position, closer to the triplet-producing carbonyl functionality, in benzaldehyde increases SOC on the single-molecule level, which enhances both the rates of ISC *k*_ISC_ and phosphorescence *k*_Phos_ significantly by 5–15 fold.^40^

Inspired by these findings as well as other successful demonstration of halogen-bond-induced phosphorescence in the solid state, we exploited the naphthalene scaffold, a prototypical building block in organic optoelectronics, to study the effect of the halogen substitution and the role of halogen bonding in mediating triplet formation. Compared to the previously studied bromobenzaldehydes, this system is expected to have less carbonyl-originated n–π* character in the low singlet excited states to drive ISC, thus offering a platform to highlight the halogen effects. Well-developed synthetic methodologies^42–45^ were used to introduce multiple halogen-bond donors (*e.g.* Br) and acceptors (*e.g.* O) in naphthalene to permit multiple non-covalent interactions to occur synergistically, enabling phosphorescence from halogen-bonded frameworks.^46^

## Results and discussion

**Scheme 1:**
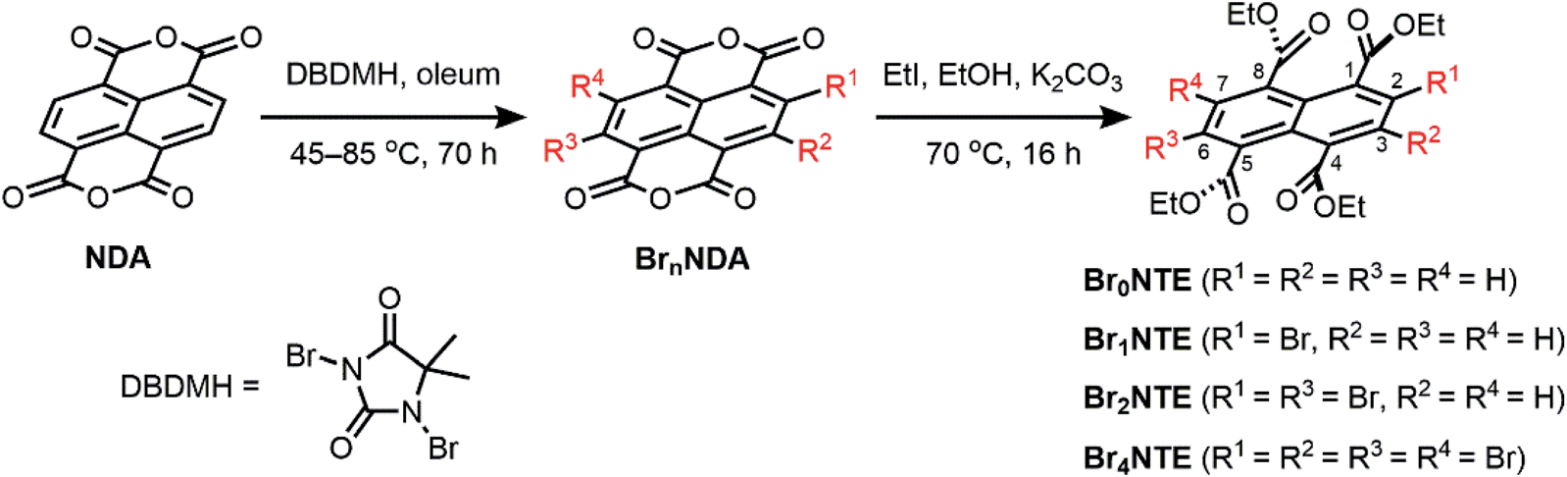
Synthesis of brominated naphthalene tetracarboxylic ethyl ester (Br*_n_*NTE).

Naphthalene derivatives with halogen-bond accepting carbonyl functionalities and a various number of halogen-bond donating Br atoms can be prepared readily from 1,4,5,8-naphthalenetetracarboxylic dianhydride (NDA) (Scheme 1).^47–49^ Bromination of NDA with 1,3-dibromo-5,5-dimethylhydantoin is slow and can produce a mixture of NDA with various numbers of Br substituents.^47^ However, the application of excess reagents at elevated temperatures for a prolonged reaction time gives tetrabrominated NDA (Br_4_NDA) as the sole product. Esterification of Br*_n_*NDA with ethyl iodide in alkaline ethanol gave a mixture of naphthalene tetracarboxylic ethyl esters, Br*_n_*NTE (*n* = 0, 1, 2, 4; *n* = 3 can be isolated but it is not discussed here for simplicity).^50,51^ The individual compounds were isolated by column chromatography on SiO_2_ and their identity was confirmed by NMR, MS, and single-crystal X-ray crystallography.

**Fig. 1:**
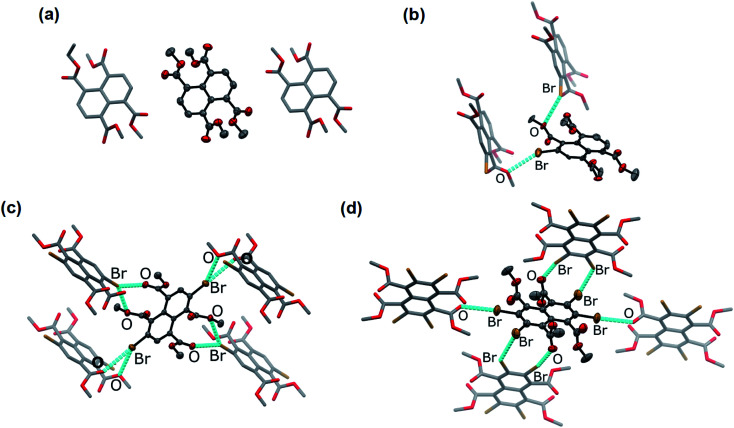
Single crystal X-ray molecular structure of (a) Br_0_NTE (space group *P*2_1_/*n*), (b) Br_1_NTE (*P*2_1_), (c) Br_2_NTE (*P*2_1_/*n*), and (d) Br_4_NTE (*P*1̄) and the close neighbours in crystals. Crystals were obtained by diffusing MeOH vapour into the CHCl_3_ solutions of Br*_n_*NTE. Colour code: C = grey, O = red, Br = brown. For Br_1_NTE, only the major component of the disorder is shown and discussed. The terminal carbon of the ethyl group and all hydrogen atoms are omitted for clarity. Thermal ellipsoids of the central molecules are shown at the 50% probability level, whereas the neighbouring molecules shown in stick representation. Non-covalent Br⋯O and Br⋯Br interactions are highlighted with cyan dashed lines.

In the solid state, an extended Br⋯O network can be observed for Br_2_NTE^50^ (Fig. 1). For each molecule, each pair of the *peri* ester groups interacts with a Br atom of a neighbouring molecule to establish bifurcated, slightly asymmetric halogen bonds with *d*(C–Br⋯O=C) = 3.268(2) Å, *d*(C–Br⋯O–C_2_H_5_) = 3.308(2) Å and both *θ*(C–Br⋯O) ∼150° (*i.e.* 152.40(9) and 149.21(8)).^38^ Reciprocally, each Br atom is interacting with two *peri* ester groups of a nearby molecule of Br_2_NTE. Being symmetrically substituted with four Br and four ester functionalities, Br_4_NTE is also embedded in a framework of halogen bonds in the solid state. However, likely due to the steric requirement of large Br atoms, the same arrangement in Br_2_NTE was not observed. Instead, only two out of the four esters on the 1 and 5 positions form linear C–Br⋯O=C short contacts with the Br atoms on the 3 and 7 positions of the neighbouring molecules (values taken from two crystallographically independent molecules: *d*(Br⋯O) = 3.074(3) and 3.286(3) Å, *θ*(C–Br⋯O) = 165.5(1) and 168.5(1)°). The remaining two Br atoms on the 2 and 6 positions are engaged in orthogonal (“Type II”)^52,53^ C–Br⋯Br–C interactions (*d*(Br⋯Br) = 3.692(1) Å, *θ*(C–Br⋯Br) = 86.4(1)°).

With only one Br atom per molecule, the ester groups in Br_1_NTE do not engage in extended halogen-bonded networks. In fact, the shortest *d*(C–Br⋯O–C_2_H_5_) distance is measured to be 3.32(2) Å (*θ*(C–Br⋯O) = 151.7(9)°), barely shorter than the van der Waals contact distance. At last, no π-stack or short contact between C–H and naphthalene was found in the crystals of Br_0_NTE.

**Fig. 2:**
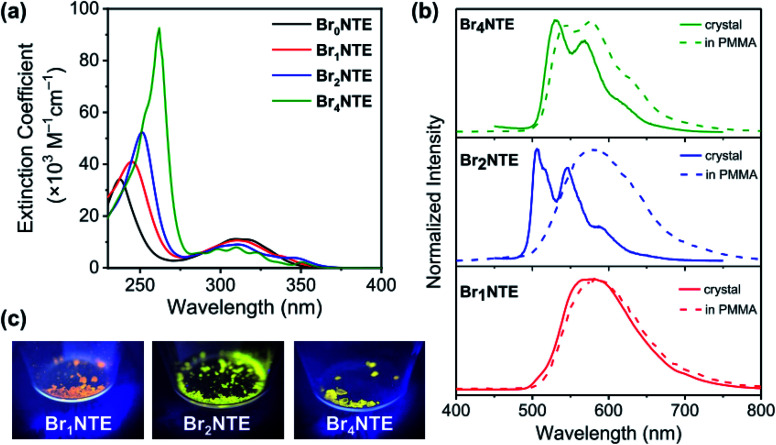
(a) UV-Vis absorption spectra of Br*_n_*NTE at (5–8) × 10^−5^ M in CH_2_Cl_2_. (b) Normalised phosphorescence spectra of Br*_n_*NTE in the crystalline solid state (solid line) or in PMMA (dashed line). Samples were excited at 300–320 nm. (c) Photographs of solid emission under UV irradiation (365 nm).

While naphthalene and its 2-brominated derivatives display electronic absorption <300 nm, ester substitution induces a bathochromic shift of the naphthalene-centred transitions by *ca.* 50 nm, extending the absorption bands to 350 nm with maxima at ∼300 nm (Fig. 2).^54–56^ Compared to pristine naphthalene, which has an appreciable fluorescence quantum yield of 23% (40% triplet formation yield),^55^ no emission was detected from all Br*_n_*NTE in deaerated CH_2_Cl_2_ up to 0.02 M (near saturation) excited at 330 nm.

Despite the non-radiative energy dissipation in solution, crystalline solids of the brominated molecules display visible phosphorescence in the 500–700 nm region with millisecond lifetimes, whereas non-brominated Br_0_NTE remains non-emissive (Fig. 2 and Table 1). Powdery crystalline solid samples of Br*_n_*NTE, whose powder X-ray diffraction profiles match the pattern based on their single-crystal data, were used in all phosphorescence measurements. Phosphorescence of crystalline Br_2_NTE and Br_4_NTE feature clear vibrational progression with a quantum yield of *Φ*_Phos_ = 19.6% and 9.3%, respectively. Much weaker and structureless emission was observed for Br_1_NTE (*Φ*_Phos_ = 1.4%).

**Table 1:** Photophysical properties of Br_*n*_NTE

	In CH_2_Cl_2_		Crystalline solids	
	*τ*(S_1_ → T_*n*_)^a^	*τ*(T_1_ → S_0_)^a^	*τ*_Phos_ b	*Φ*_Phos_ b ^,^ c
Br_0_NTE	111.7 ± 0.8 ps^d^	29.1 ± 0.1 μs	n.a.	n.a.
Br_1_NTE	9.1 ± 0.3 ps^d^	1.42 ± 0.01 μs	1.53 ± 0.02 ms	1.4%
Br_2_NTE	7.5 ± 0.3 ps	0.50 ± 0.01 μs	1.94 ± 0.01 ms	19.6%
Br_4_NTE	48.3 ± 0.9 ps^d^	0.0121 ± 0.0006 μs	1.11 ± 0.01 ms	9.3%

a From transient absorption measurements.

b From (time-resolved) phosphorescence measurements.

c The uncertainty is estimated to be 20% of the measured values.

d Preceded by the relaxation of hot S_1_ in (0.9–1.2)±0.3 ps.

The varying luminescent behaviours suggest that the excited-state dynamics were modulated in a subtle way by Br-specific properties, which is however not directly related to the number of Br atoms in the molecule. It is conceivable that multi-point halogen bonding provides a geometric framework to strengthen the rigidity of Br*_n_*NTE in the crystalline state. This effect is especially substantial for Br_2_NTE where all the peripheral substituents engage in the directional Br⋯O interactions, providing the additional factor to the solid-state effect^6,8^ of RTP to impede competitive non-radiative relaxation through intramolecular motions. The highest phosphorescence quantum yield was thus observed for the crystalline sample of Br_2_NTE.

The weaker and non-structured phosphorescence observed for Br_1_NTE (and Br_0_NTE) seems to be originated from its looser solid-state packing. If we define the volumetric index *V*_i_ as the ratio between the Voronoi volume (*V*_Vor_)^57,58^ and the van der Waals volume (*V*_wdW_) of a molecule in the crystal, smaller *V*_i_ = *V*_Vor_/*V*_wdW_ would suggest denser packing. *V*_i_ of 1.27–1.30 were found for Br_2_NTE and Br_4_NTE embedded in halogen-bonded frameworks, but the values are significantly larger for Br_0_NTE and Br_1_NTE (1.36–1.38). The larger free space available to each molecule in the Br_0_NTE and Br_1_NTE crystals allows the excited molecules to decay radiatively and non-radiatively on various points of the triplet potential energy surface.

The significance of the inter-Br*_n_*NTE Br⋯O interactions is further supported by comparing the phosphorescence of crystalline Br*_n_*NTE with that of the dispersed molecules in poly(methyl methacrylate) (PMMA, *M*_w_ ∼996 kDa; 2 wt% doping). The rigid polymer matrix is expected to constrain the molecular motion at room temperature but disrupt inter-Br*_n_*NTE Br⋯O halogen bonds. The phosphorescence spectra of Br_1_NTE remained identical in either environment (Fig. 2), indicating that the triplet decay in Br_1_NTE is largely intrinsic to the monomeric molecule. However, the vibrational progression of Br_4_NTE, a signature of chromophore rigidity, became less pronounced, and that of Br_2_NTE completely disappeared and the overall emission profile resembles very well to that of Br_1_NTE.

**Fig. 3:**
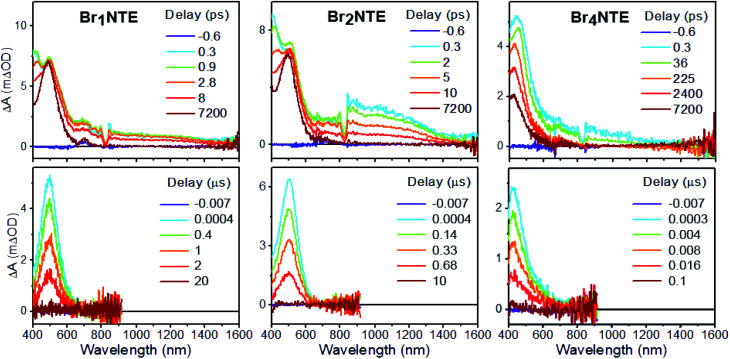
Transient absorption spectra of Br*_n_*NTE (*n* = 1, 2, and 4) in deaerated CH_2_Cl_2_ at various pump–probe delay indicated (excitation = 330 nm, see ESI Section 5† for *n* = 0).

Additional support for the efficient population of the triplet excited state was provided by transient absorption measurements. Spectroscopically, all Br*_n_*NTE exhibit similar excited-state dynamics: following the initial formation of the singlet excited state, which displays excited-state absorption (ESA) peaking at *ca.* 490 nm and a broad feature in the near infrared region of 800–1000 nm (Fig. 3 and ESI Section 5†), a new excited-state species with ESA at *ca.* 480 nm appears with microsecond lifetimes. This long-lived species was assigned to the triplet of each chromophore based on the lifetime and spectral similarity to the triplet–triplet absorption of methyl 1-naphthalate^59^ and 2-bromonaphthalene.^60^ Therefore, the decay of the initial state can be ascribed to singlet-to-triplet ISC; time constants on the order of tens of picosecond were observed for this process (Table 1). Compared to the typical fluorescence lifetime (1 ns or longer) of naphthalene derivatives,^54,61^ the fast ISC process suggests a high triplet forming efficiency. Such efficient ISC on the molecular level is likely due to the combined results of bromo (*cf.* >90% triplet yield for 2-bromonaphthalene)^60^ and carbonyl substitution.^62^ Broadly speaking, the more bromo atoms in a molecule, the faster the S_1_ → T_*n*_ and T_1_ → S_0_ processes, in line with the stronger heavy-atom enhanced SOC.^63^ Unexpectedly, however, the S_1_ → T_*n*_ ISC for Br_4_NTE is noticeably slower than its less brominated analogues.

Since the rate of S_1_ → T_*n*_ ISC is largely determined by the energy gap between the singlet and triplet states (Δ*E*_ST_) and the magnitude of spin–orbit coupling (SOC),^64^ we evaluated the matrix elements of 〈S_1_|*Ĥ*_SO_|T_*n*_〉 using the two-layer ONIOM (QM:MM) scheme to simulate the photophysical processes in crystals. The molecular geometry was computed at the level of ωB97X-D/6-31G(d):OPLS-AA, and 〈S_1_|*Ĥ*_SO_|T_*n*_〉 calculated at the TDA-ωB97X-D/6-311+G(d,p) level of theory based on the ONIOM geometries (Table 2 and ESI Section 7†). The (TD-)DFT calculations were performed using Gaussian 16,^65^ which was then interfaced with PySOC^30^ to evaluate the SOC matrix elements. The Tamm–Dancoff approximation (TDA) was exploited to minimise triplet instability.^40,66^ In all cases, the state energies are not significantly affected by aggregation; thus results from the calculations with one molecule in the QM region are discussed here.

**Table 2:** Spin–orbit coupling (in cm^−1^) calculated at the TDA-ωB97X-D/6-311+G(d,p) level of theory based on the ONIOM geometries

	At S_1_ geometry^a^				At T_1_ geometry
	〈S_1_|*Ĥ*_SO_|T_1_〉	〈S_1_|*Ĥ*_SO_|T_2_〉	〈S_1_|*Ĥ*_SO_|T_3_〉	〈S_1_|*Ĥ*_SO_|T_4_〉	〈S_0_|*Ĥ*_SO_|T_1_〉
Br_0_NTE	0.82	0.04	0.52	0.86	0.01
Br_1_NTE	8.22	**10.87**	**15.49**	9.02	3.22
Br_2_NTE	68.74	0.93	**166.79**	**106.08**	142.3
Br_4_NTE	20.30	**42.79**	3.55	30.93	0.38

a States relevant for the intersystem-crossing mechanism are highlighted in bold.

The ISC process for Br*_n_*NTE likely takes place between S_1_ and the high-lying triplet states. Considering Δ*E*_ST_ alone (<0.5 eV), ISC to T_2,3_ for Br_1_NTE, T_2–4_ for Br_2_NTE, and T_2_ for Br_4_NTE should dominate in the respective molecules, whereas the large energy gap Δ*E*_ST_ > 1.5 eV prevents direct ISC into T_1_ (see ESI Section 7† for the relative energies). Compared to Br_0_NTE, incorporating fourth-row Br elements into the naphthalene scaffold increases SOC by 1–2 orders of magnitude. Despite the larger number of Br atoms in the structure, smaller SOC was found for Br_4_NTE than Br_2_NTE, in line with the slower triplet formation found experimentally for the former molecule. The 〈S_0_|*Ĥ*_SO_|T_1_〉 calculated at the T_1_ geometry, the key factor determining the rate of phosphorescence, was similarly found to be smaller for Br_4_NTE than Br_2_NTE.

**Fig. 4:**
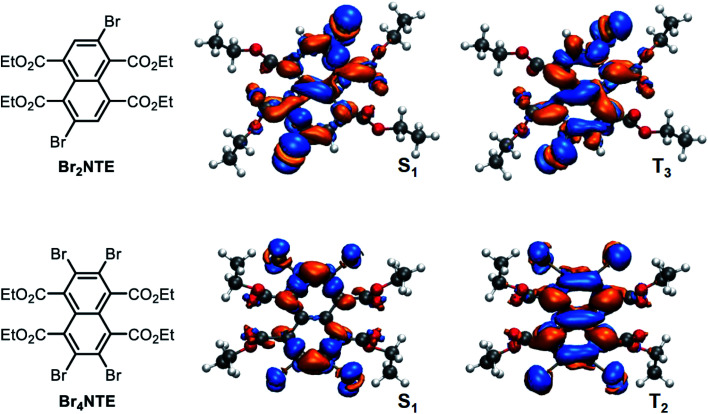
Top-down view of electron density difference plots (0.001 e bohr^−3^ isovalue) between the selected excited states (S_1_ or T_2/3_) and the ground state for Br_2_NTE (top row) and Br_4_NTE (bottom row). The molecular orientation is sketched on the left; orange colour represents positive and blue negative values.

A close examination of the electron density of the key states provided hints to the origin of the unexpected drop in SOC for Br_4_NTE. Fig. 4 shows the electron density difference between the selected excited states and the ground state for Br_2_NTE (S_1_ and T_3_) and for Br_4_NTE (S_1_ and T_2_). These transitions displayed a significant naphthalene-centred π–π* character; the involvement of the Br atoms can be clearly seen and hence the higher SOC in brominated Br*_n_*NTE. Comparatively, the carbonyl n–π* contribution, the typical driver for the ISC process in aromatic ketones/aldehydes, appears to be much less substantial. In the case of Br_2_NTE, the Br-centred transition densities are roughly perpendicular to the naphthalene plane in the S_1_ state but rotate distinctively in the T_3_ state, facilitating the orbital angular momentum change for ISC (similar rotation found in T_4_). In the case of Br_4_NTE, however, the Br-centred transition densities in S_1_ and T_2_ are both perpendicular to the naphthalene plane. The absence of the analogous rotated transition density for Br_4_NTE is understandable as unfavourable electron repulsion in the region between neighbouring Br atoms would be caused by such a change in density orientation.

Taken together, the judicious heavy-atom positioning in Br_2_NTE results in the favourable structural and electronic contributions to its efficient RTP. The 2,6-dibromo substitution offers a lock-in mechanism through halogen bonding to inhibit non-radiative relaxation. Furthermore, high SOC and hence efficient ISC are made possible by adding the orbital angular momentum change to the heavy-atom effect in both the triplet-generation (S_1_ → T_*n*_) and phosphorescence (T_1_ → S_0_) processes.

## Conclusions

In summary, we have shown that simultaneously incorporating multiple heavy halogens and halogen-bond donor/acceptor pairs in aromatic molecules can enable bright phosphorescence from purely organic materials. The formation of halogen-bonded frameworks in the solid states rigidifies phosphorophores, favouring the radiative decay. However, our results indicate that a fine balance has to be struck in terms of the number and positioning of halogens. Too many large halogen atoms in proximity may prohibit *structurally* the access of halogen-bond acceptors and *electronically* the contribution of the non-bonding electrons of halogens for enhancing SOC. The latter effect is especially important to consider in the case of carbonyl-bearing polycyclic aromatic hydrocarbons, such as rylenes and its derivatives in the present study where the S_1_ state is primarily π–π* in nature. It should be noted that the formation of halogen bonds cannot necessarily be correlated to the increase in ISC and phosphorescence rates; an excited-state analysis will be needed to elucidate the magnitude and origin of SOC when designing organic RTP materials.

## Conflicts of interest

There are no conflicts to declare.

## Supplementary Material

SC-012-D0SC04646A-s001

SC-012-D0SC04646A-s002
