# Supplementary material for: Organic room-temperature phosphorescence from halogen-bonded organic frameworks: hidden electronic effects in rigidified chromophores
Source: Chem Sci. 2020 Nov 5;12(2):767–73. doi: 10.1039/d0sc04646a (PMC8178982; doi:10.1039/d0sc04646a)
Supplement: SC-012-D0SC04646A-s001 [file SC-012-D0SC04646A-s001.pdf]

Electronic Supplementary Material (ESI) for Chemical Science.
This journal is © The Royal Society of Chemistry 2020

Electronic supplementary information

Organic room-temperature phosphorescence from halogen-bonded organic frameworks: hidden electronic effects in rigidified chromophores

Jiawang Zhou,[a] Ljiljana Stojanović,[b] Andrey A. Berezin,[c] Tommaso Battisti,[c] Abigail Gill,[c] Benson M. Kariuki,[c] Davide Bonifazi,[c,d] Rachel Crespo-Otero,*[b] Michael R. Wasielewski*[a] and Yi-Lin Wu*[c]

[a]*Department of Chemistry and Institute for Sustainability and Energy at Northwestern, Northwestern University, Evanston, Illinois 60208-3113, United States. E-mail: m-wasielewski@northwestern.edu*
[b]*School of Biological and Chemical Sciences, Queen Mary University of London, London E1 4NS, United Kingdom. E-mail: r.crespo-otero@qmul.ac.uk*
[c]*School of Chemistry, Cardiff University, Cardiff CF10 3AT, United Kingdom. E-mail: WuYL@cardiff.ac.uk*
[d]*Institute of Organic Chemistry, Faculty of Chemistry, University of Vienna, Währinger Str. 38, 1090, Vienna, Austria.*

Table of Contents

1. Experimental and instrumentation 2
2. Synthetic details 4
3. Single-crystal structural characterisation 6
4. Powder X-ray diffraction characterisation 9
5. Transient absorption analysis 10
6. Excitation spectra 20
7. Computation details 22
8. AIM analysis of C–Br···OEt and C–Br···O=C interactions 29
9. Temperature-dependent phosphorescence 30
10. ^{1}H and ^{13}C NMR Spectra 31
11. References 34

1. Experimental and instrumentation

UV-Vis Spectroscopy. Solution phase absorption spectra were recorded on a Shimadzu 1800 spectrophotometer.

Photoluminescence spectroscopy. Photoluminescence measurements were performed at room temperature using a HORIBA Nanolog spectrofluorimeter equipped with a 450-W Xe lamp excitation source and a photomultiplier tube (PMT) detector. Absolute photoluminescence quantum yields were determined using an integrating sphere (Horiba Quanta–φ). Time-resolved experiments were carried out with a time-correlated single-photon counting (TCSPC) module, and the excitation wavelength is 355 nm. Phosphorescence emission was collected with a 3 nm increment.

Powder X-ray diffraction (PXRD) patterns were measured on a Rigaku ATX-G diffractometer or a Bruker D8 instrument (Cu Kα) in the transmission mode. The powder samples were either placed in a thin-walled glass capillary tube (Charles-Supper, 0.7 mm o.d.) or held between two pieces of Scotch tape.

Single-crystal diffraction. Single crystals were grown by slow diffusion of MeOH vapour into the $CHCl_3$ solution of the sample. X-ray diffraction data were collected on a Rigaku Oxford diffraction SuperNova Dual Atlas diffractometer with a mirror monochromator using Mo-Kα radiation. The crystal was kept at 293 K during data collection. The structures were solved with the ShelXT[1] structure solution program using Intrinsic Phasing and refined with the ShelXL[2] refinement package using Least Squares minimisation using Olex2.[3]

The structure of **Br_1NTE** is disordered through a pseudo-inversion centre located at the centre of the central C–C bond of the fused rings. As a result, the Br atom is disordered over two sites which are shared with a hydrogen atom. The two sites have occupancies of 0.686 and 0.314 for the Br and the inverse for H. Non-hydrogen atoms were refined with anisotropic displacement parameters. Hydrogen atoms were placed in idealised positions, and a riding model was used with U_{iso}(H) set at 1.2 or 1.5 times the value of U_{eq} for the atom to which they are bonded.

Transient absorption. Femtosecond and nanosecond transient absorption (TA) experiments were performed using an instrument previously described.[4] The 330 nm, ~60 fs pump pulses were depolarised using a commercial depolariser (DPU-25-A, Thorlabs, Inc.) to eliminate any orientational dynamics contributions from the experiment. Spectra were collected on commercial spectrometers for each time window (customised Ultrafast Systems, LLC Helios

and EOS spectrometers, for fsTA and nsTA, respectively). All samples were deaerated and prepared in a N_2 glove box in a 2 mm path length cell and were stirred to avoid localised heating or degradation effects during optical measurements. Based on the extinction coefficients, we estimated that the sample concentrations used in the time-resolved absorption experiments to be around 8×10^{-4} M. Intermolecular interactions between **Br$_n$NTE** are negligible in such a concentration range given the identical normalised UV-Vis spectra recorded at different concentrations.

PMMA dispersed chromophores. Br$_n$NTE (0.8–1 mg) and PMMA (40 mg) were dissolved in $CHCl_3$ (1.5 mL) with ultrasonication for 5 min at room temperature in a 4-mL shell vial. After mixing, $CHCl_3$ was let evaporated at room temperature, leaving a thin layer of PMMA at the bottom of the shell vial. The residual solvent was further removed by leaving the vial under vacuum (0.02 mbar) overnight. The free-standing thin film **Br$_n$NTE** in PMMA (~ 2 wt%) was used in phosphorescence characterisation.

2. Synthetic details

Materials and general methods. Reagents were purchased at the reagent grade and used as received. Flash column chromatography was carried out with SiO_2 (particle size 0.040–0.063 mm, 230–400 mesh) and technical solvents. ^1H and ^{13}C NMR spectra were measured on a Bruker AVANCE III HD 500 instrument. Chemical shifts were reported in ppm relative to the signal of $Si(CH_3)_4$ using the solvent residual signal as an internal reference ($CDCl_3$: δ_H = 7.26 ppm, δ_C = 77 ppm). Coupling constants (*J*) were given in Hz. The apparent resonance multiplicity was described as s (singlet), d (doublet), t (triplet), q (quartet) and m (multiplet). Infrared spectra (IR) were recorded on a Shimadzu IR Affinity 1S FTIR spectrometer with a single reflection ATR accessory (Specac, diamond crystal); vibration modes are reported in cm^{-1}. High-resolution mass spectra (HR-MS) were performed on a Waters LCT HR TOF mass spectrometer; signals are reported in m/z units.

General Procedure A for the preparation of brominated naphthalene-1,4,5,8-tetracarboxylic dianhydride (Br_nNDA).[5-8] Naphthalene-1,4,5,8-tetracarboxylic dianhydride (NDA, 1609 mg, 6 mmol) was dispersed in concentrated sulfuric acid (95–98%, 80 mL for the synthesis of a mixture of **Br_nNDA**) or fuming sulfuric acid (20% free SO_3, 80 mL for **Br_4NDA**), and the mixture was stirred at room temperature (r.t.) for 5 min in a heavy-wall, round-bottom pressure flask. An appropriate amount of 1,3-dibromo-5,5-dimethylhydantoin (1.5 equiv. for the synthesis of a mixture of **Br_nNDA** or and 4 equiv. for **Br_4NDA**) was then added in 5 portions at r.t. over 30 min. The flask was sealed and heated at 45 or 85 ºC for **Br_n-** and **Br_4NDA**, respectively for 70 h. *CAUTION: Bromine vapour accumulated*. The reaction mixture was poured over crushed ice and filtered; the yellow precipitate was washed with water and methanol and dried under vacuum. The product (bright yellow solids) was used in the esterification reaction without further purification.

General Procedure B for the preparation of brominated tetraethyl naphthalene-1,4,5,8-tetracarboxylate (Br_nNTE).[5, 8, 9] **Br_nNDA** (n=0–4, 2 mmol, 1 equiv.) was placed in a Schlenk flask with potassium carbonate (1658 mg, 12 mmol, 6 equiv.), ethyl iodide (2.5 mL) and EtOH (10 mL). The flask was sealed, and the mixture was stirred at 70 ºC overnight. The organic layer was separated with water and CH_2Cl_2, and dried over Na_2SO_4. The solvents were evaporated under vacuum and product purified by column chromatography on SiO_2 with a solvent gradient from CH_2Cl_2:hexanes 4:1 to 100% CH_2Cl_2. Compounds (white solids) were eluted out and collected subsequently from n = 4 (shortest retention time) to n = 0 (longest

retention). The ^{1}H NMR, ^{13}C NMR, and HR-ESI-MS data (see below) are consistent with the expected or reported (**Br$_2$**- and **Br$_4$NTE**)[5, 8] structure.

Tetraethyl naphthalene-1,4,5,8-tetracarboxylate (Br$_0$NTE). ^{1}H NMR (500 MHz, $CDCl_3$): δ = 1.39 (t, J = 7.2 Hz, 12 H; CH_3), 4.37 (q, J = 7.2 Hz, 8 H; CH_2), 8.04 (s, 4 H; Ar-H) ppm; ^{13}C NMR (125 MHz, $CDCl_3$): δ = 14.05, 61.74, 128.93, 129.00, 133.53, 167.92 ppm; IR (ATR): $\tilde{\nu}$ = 2992 (w, C-H), 1717 (s, C=O), 1583 (w), 1477 (w), 1460 (w), 1443 (w), 1383 (w), 1362 (w), 1333 (w), 1277 (s), 1252 (s), 1200 (w), 1173 (m) 1150 (s), 1113 (w), 1096 (s), 1013 (s), 916 (w), 880 (m), 858 (m), 793 (w) 756 (s), 723 (m), 650 (w), 447 (w). 424 (w), 413 (w) cm^{-1}; HR-ESI-MS: 439.1366 *m/z* ([*M*+Na]$^+$, calcd for $C_{22}H_{24}O_8Na^+$: 439.1363).

Tetraethyl 2-bromonaphthalene-1,4,5,8-tetracarboxylate (Br$_1$NTE). ^{1}H NMR (500 MHz, $CDCl_3$): δ = 1.33–1.47 (m, 12 H; CH_3), 4.28–4.47 (m, 8 H), 7.90 (d, J = 7.4 Hz, 1 H; Ar-H), 8.03 (d, J = 7.4 Hz, 1 H; Ar-H), 8.21 (s, 1 H; Ar-H) ppm; ^{13}C NMR (125 MHz, $CDCl_3$): δ = 13.86, 14.03, 14.07, 61.85, 62.06, 62.18, 121.29, 127.70, 128.97, 129.15, 129.66, 133.05, 133.32, 133.48, 134.27, 134.32, 166.58, 166.64, 167.51, 167.73 ppm (2 peaks at 14.03 and 62.06 ppm each representing 2 overlapped ethyl carbons); IR (ATR): $\tilde{\nu}$ = 2978 (w, C-H), 1713 (s, C=O), 1562 (w), 1514 (w), 1470 (w, C-H), 1447 (w, C-H), 1408 (w), 1365 (w), 1346 (w), 1306 (w), 1287 (m), 1271 (m), 1250 (m), 1206 (w), 1175 (m, C-O), 1155 (m, C-O), 1092 (m, C-O), 1016 (m), 932 (w), 918 (w), 860 (m), 789 (w), 758 (m), 727 (w), 684 (w), 644 (w), 600 (w), 521 (m, C-Br), 480 (w), 447 (w), 417 (w) cm^{-1}; HR-ESI-MS: 517.0479 *m/z* ([*M*+Na]$^+$, calcd for $C_{22}H_{23}{}^{79}Br_1O_8Na^+$: 517.0469).

3. Single-crystal structural characterisation

All crystallographic data are available free of charge from the Cambridge Crystallographic Data Centre (CCDC) via www.ccdc.cam.ac.uk/data_request/cif.

Table S1. Crystal data and structure refinement for **Br_0NTE.**

Empirical formula	$C_{22}H_{24}O_8$	
Formula weight	416.41	
Temperature	293(2) K	
Crystal system	monoclinic	
Space group	$P2_1/n$	
Unit cell dimensions	a = 8.5666(7) Å	α = 90°
	b = 14.3257(7) Å	β = 112.196(10)°
	c = 9.2306(8) Å	γ = 90°
Volume/	1048.86(15) Å^3	
Z	2	
Density (calculated)	1.319 g/cm^3	
Absorption coefficient	0.101 mm^{-1}	
F(000)	440	
Crystal size	0.249 × 0.207 × 0.163 mm^3	
Radiation	MoKα (λ = 0.71073)	
2Θ range for data collection	7.422° to 59.492°	
Index ranges	$-11 \leq h \leq 11$, $-19 \leq k \leq 19$, $-12 \leq l \leq 12$	
Reflections collected	8629	
Independent reflections	2519 [R_{int} = 0.0206, R_{sigma} = 0.0211]	
Data/restraints/parameters	2519/0/138	
Goodness-of-fit on F^2	1.061	
Final R indexes [I>=2σ (I)]	R_1 = 0.0779, wR_2 = 0.2365	
Final R indexes [all data]	R_1 = 0.1009, wR_2 = 0.2609	
Largest diff. peak/hole	0.71/-0.31 e Å^{-3}	
CCDC Number	1949875	

Table S2. Crystal data and structure refinement for **Br_1NTE.**

Empirical formula	$C_{22}H_{23}BrO_8$	
Formula weight	495.31	
Temperature	293(2) K	
Crystal system	monoclinic	
Space group	$P2_1$	
Unit cell dimensions	a = 9.6364(5) Å	α = 90°
	b = 9.8092(5) Å	β = 90.442(4)°
	c = 11.8602(5) Å	γ = 90°
Volume	1121.06(9) $Å^3$	
Z	2	
Density (calculated)	1.467 g/cm^3	
Absorption coefficient	1.878 mm^{-1}	
F(000)	508	
Crystal size	0.193 × 0.139 × 0.102 mm^3	
Radiation	MoKα (λ = 0.71073)	
2Θ range for data collection	6.834° to 59.084°	
Index ranges	$-12 \leq h \leq 13$, $-9 \leq k \leq 13$, $-16 \leq l \leq 14$	
Reflections collected	10647	
Independent reflections	4142 [R_{int} = 0.0332, R_{sigma} = 0.0372]	
Data/restraints/parameters	4142/1/294	
Goodness-of-fit on F^2	1.046	
Final R indexes [I>=2σ (I)]	R_1 = 0.0677, wR_2 = 0.1502	
Final R indexes [all data]	R_1 = 0.0863, wR_2 = 0.1610	
Largest diff. peak/hole	0.41/-0.54 e $Å^{-3}$	
Flack parameter	0.339(8)	
CCDC number	1949880	

Table S3. Crystal data and structure refinement for **Br_4NTE.**

Empirical formula	$C_{22}H_{20}Br_4O_8$	
Formula weight	732.02	
Temperature/K	293(2) K	
Crystal system	triclinic	
Space group	P-1	
Unit cell dimensions	a = 9.5351(5) Å	α = 72.585(5)°
	b = 12.0414(6) Å	β = 69.310(5)°
	c = 12.8133(8) Å	γ = 89.363(4)°
Volume	1305.80(14) $Å^3$	
Z	2	
Density (calculated)	1.862 g/cm^3	
Absorption coefficient	6.207 mm^{-1}	
F(000)	712	
Crystal size	0.25 × 0.131 × 0.084 mm^3	
Radiation	MoKα (λ = 0.71073)	
2Θ range for data collection	6.784° to 59.71°	
Index ranges	$-13 \le h \le 11$, $-15 \le k \le 16$, $-16 \le l \le 12$	
Reflections collected	10370	
Independent reflections	6162 [R_{int} = 0.0212, R_{sigma} = 0.0434]	
Data/restraints/parameters	6162/0/311	
Goodness-of-fit on F^2	1.020	
Final R indexes [I>=2σ (I)]	R_1 = 0.0426, wR_2 = 0.0802	
Final R indexes [all data]	R_1 = 0.0773, wR_2 = 0.0937	
Largest diff. peak/hole	0.75/-0.74 e $Å^{-3}$	
CCDC number	1949883	

4. Powder X-ray diffraction characterisation

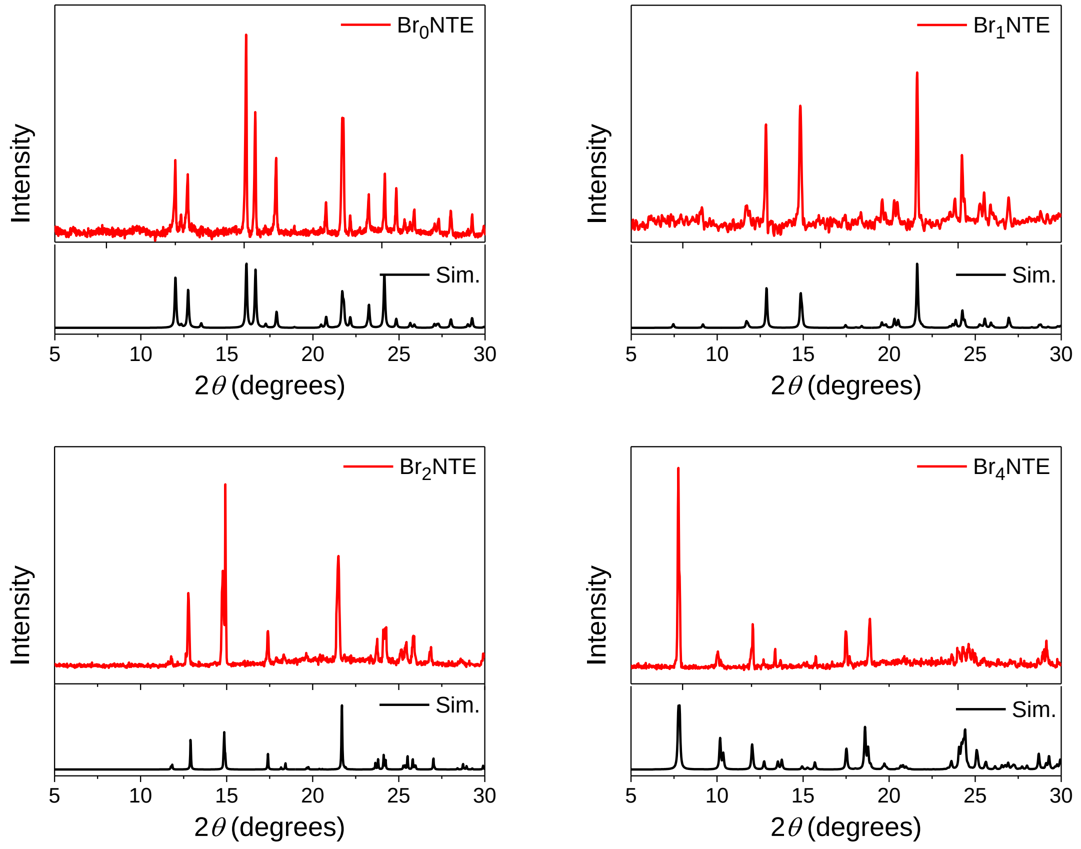

Figure S1. Comparison of the powder X-ray diffraction profiles (red line) of the solid samples used in phosphorescence measurements with the corresponding simulated pattern (black line) based on the single-crystal data. The slight mismatch in relative peak intensities is originated from preferred orientation present in the powder samples.

5. Transient absorption analysis

All transient absorption data were background-subtracted to remove scattered light and thermally lensed fluorescence from the spectra, and then corrected for group delay dispersion (GDD, or “chirp”) using Surface Xplorer Pro 4 (Ultrafast Systems, LLC). Kinetic traces were fit (singly or globally) in a laboratory-written MATLAB program. The program solves the differential equations of the specified kinetic model, and then convolutes them with a Gaussian instrument response function before employing a least-squares fitting to iteratively find the parameters which result in matches to the same functions for all selected wavelengths. Once these parameters are established, they are fed into the model solutions to yield the populations of each state in model. Finally, the total raw data matrix is deconvoluted with these populations as functions of time to produce the spectra associated with each species.

We used the following first-order kinetic models with rate matrices K for the compounds and excitation wavelengths:

For the fsTA of **Br$_0$NTE** and **Br$_1$NTE**:

$$K = \begin{pmatrix} -k_1 & 0 & 0 \\ k_1 & -k_2 & 0 \\ 0 & k_2 & 0 \end{pmatrix} \qquad \text{(Eqn. S1)}$$

where k_1 and k_2 represent the nascent S_1 structural relaxation rate and singlet-to-triplet intersystem crossing rate, respectively.

For the fsTA of **Br$_2$NTE**:

$$K = \begin{pmatrix} -k_1 & 0 \\ k_1 & 0 \end{pmatrix} \qquad \text{(Eqn. S2)}$$

where k_1 represents the singlet-to-triplet intersystem crossing rate.

For the fsTA of **Br$_4$NTE**:

$$K = \begin{pmatrix} -k_1 & 0 & 0 & 0 \\ k_1 & -k_2 & 0 & 0 \\ 0 & k_2 & -k_3 & 0 \\ 0 & 0 & k_3 & 0 \end{pmatrix} \qquad \text{(Eqn. S3)}$$

where k_1 and k_3 represent the structural relaxation rates in the singlet and triplet manifold, respectively. k_2 is the singlet-to-triplet intersystem crossing rate.

For the nsTA of all compounds:

$$K = (k_1) \qquad \text{(Eqn. S4)}$$

where k_1 represents the triplet to singlet intersystem crossing rate.

- Transient absorption of **Br$_0$NTE** in CH_2Cl_2:

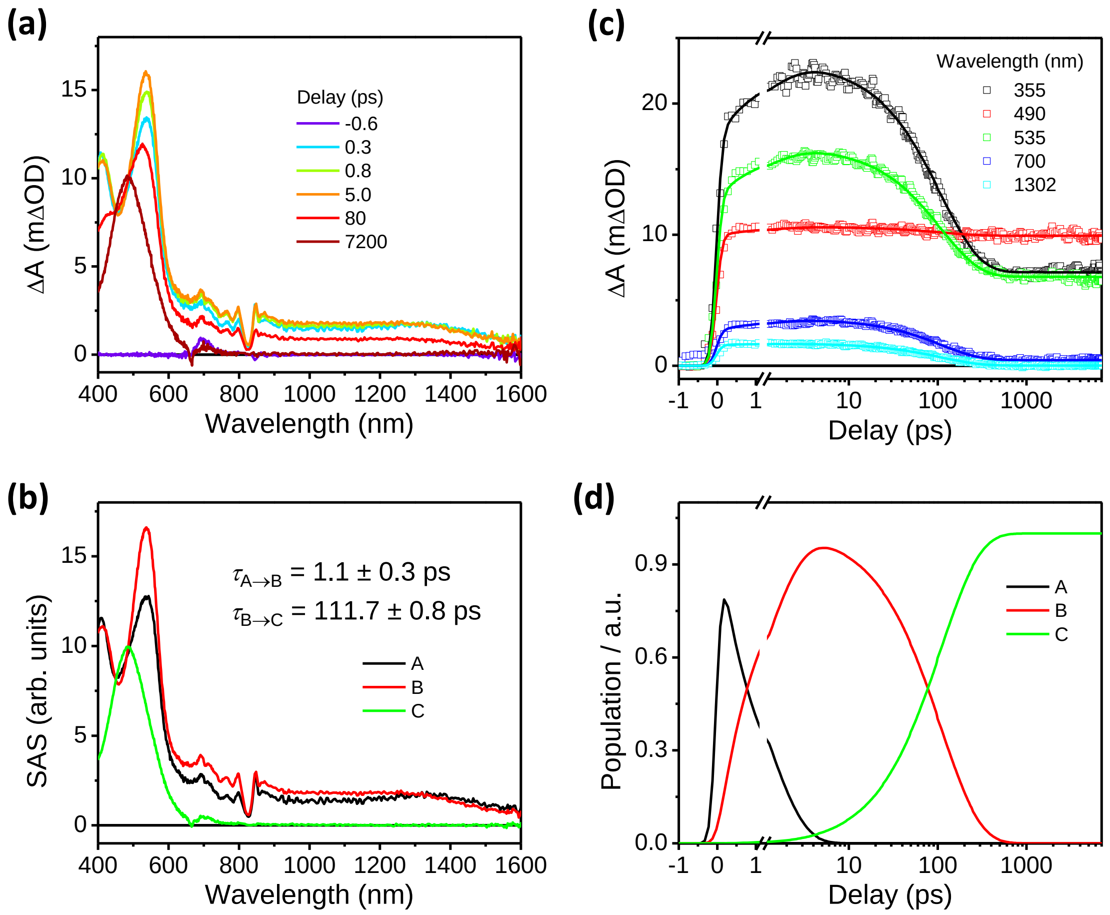

Figure S2. (a) fsTA spectra, (b) species-associated spectra (SAS), (c) multiple-wavelength fits, and (d) populations of kinetic states of **Br$_0$NTE** in deaerated CH_2Cl_2 excited at 330 nm (A: hot S_1, B: relaxed S_1, C: T_1).

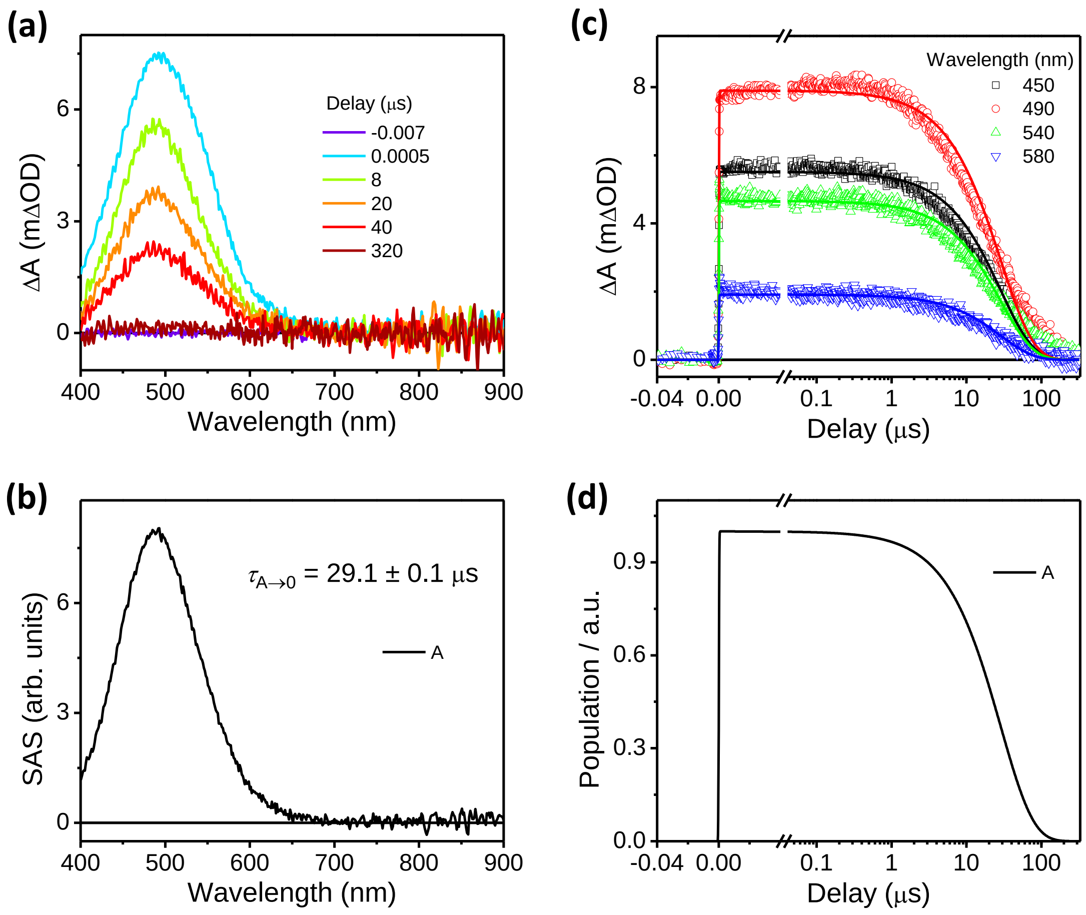

Figure S3. (a) nsTA spectra, (b) species-associated spectra (SAS), (c) multiple-wavelength fits, and (d) populations of kinetic states of **Br$_0$NTE** in deaerated CH_2Cl_2 excited at 330 nm (A: T_1).

- Transient absorption of **Br_1NTE** in CH_2Cl_2:

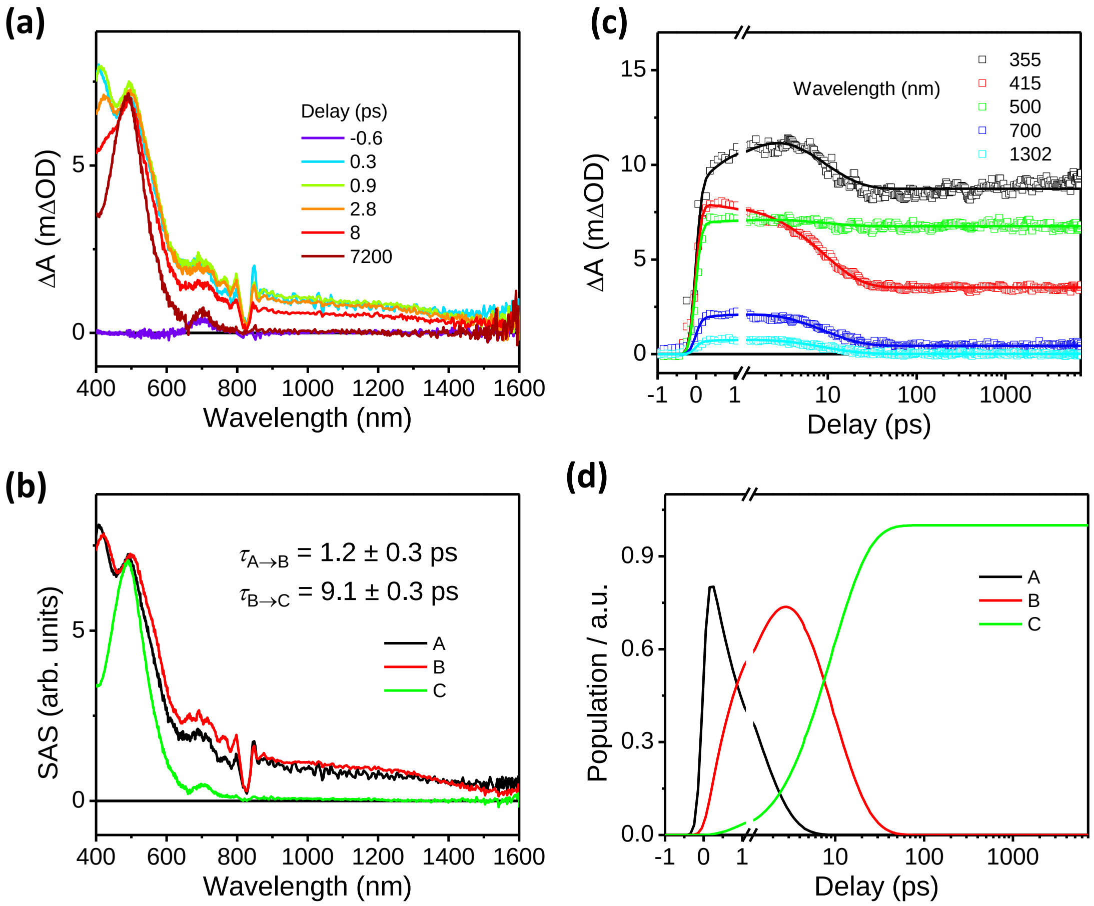

Figure S4. (a) fsTA spectra, (b) species-associated spectra (SAS), (c) multiple-wavelength fits, and (d) populations of kinetic states of **Br_1NTE** in deaerated CH_2Cl_2 excited at 330 nm (A: hot S_1, B: relaxed S_1, C: T_1).

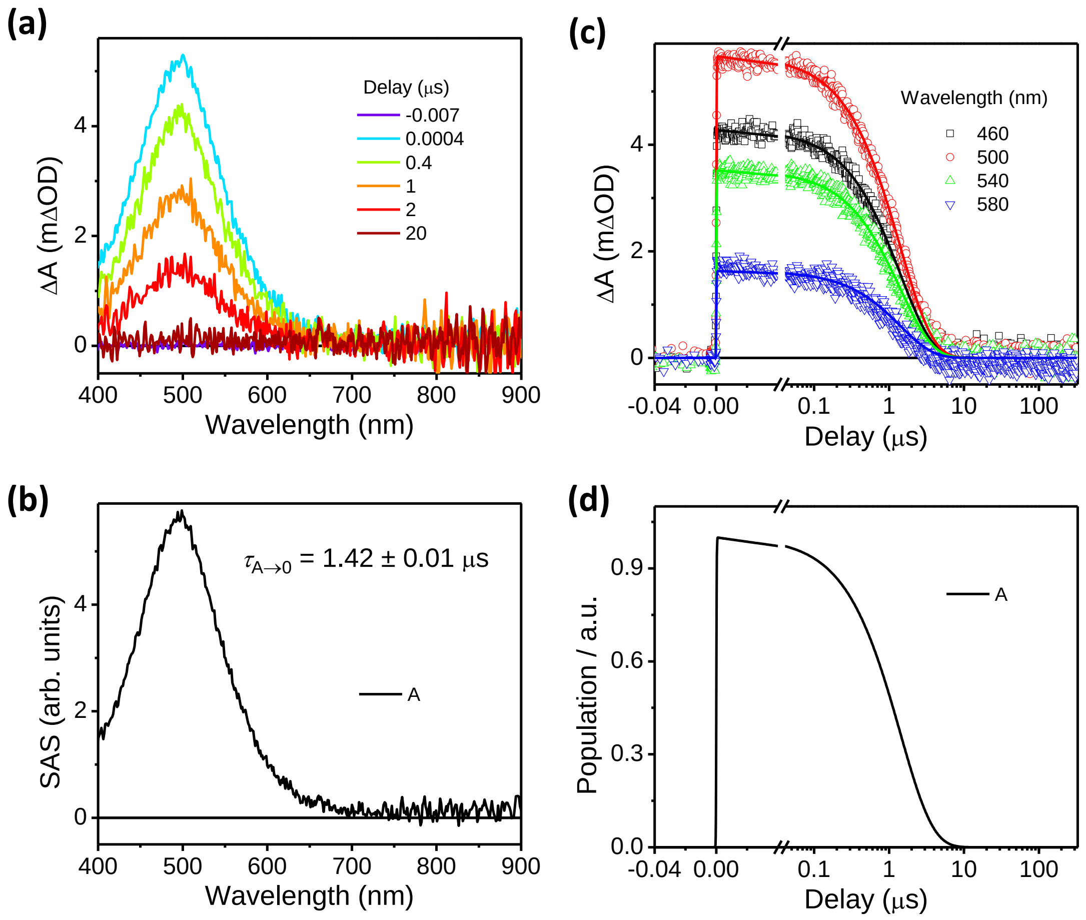

Figure S5. (a) nsTA spectra, (b) species-associated spectra (SAS), (c) multiple-wavelength fits, and (d) populations of kinetic states of **Br_1NTE** in deaerated CH_2Cl_2 excited at 330 nm (A: T_1).

- Transient absorption of **Br2NTE** in CH_2Cl_2:

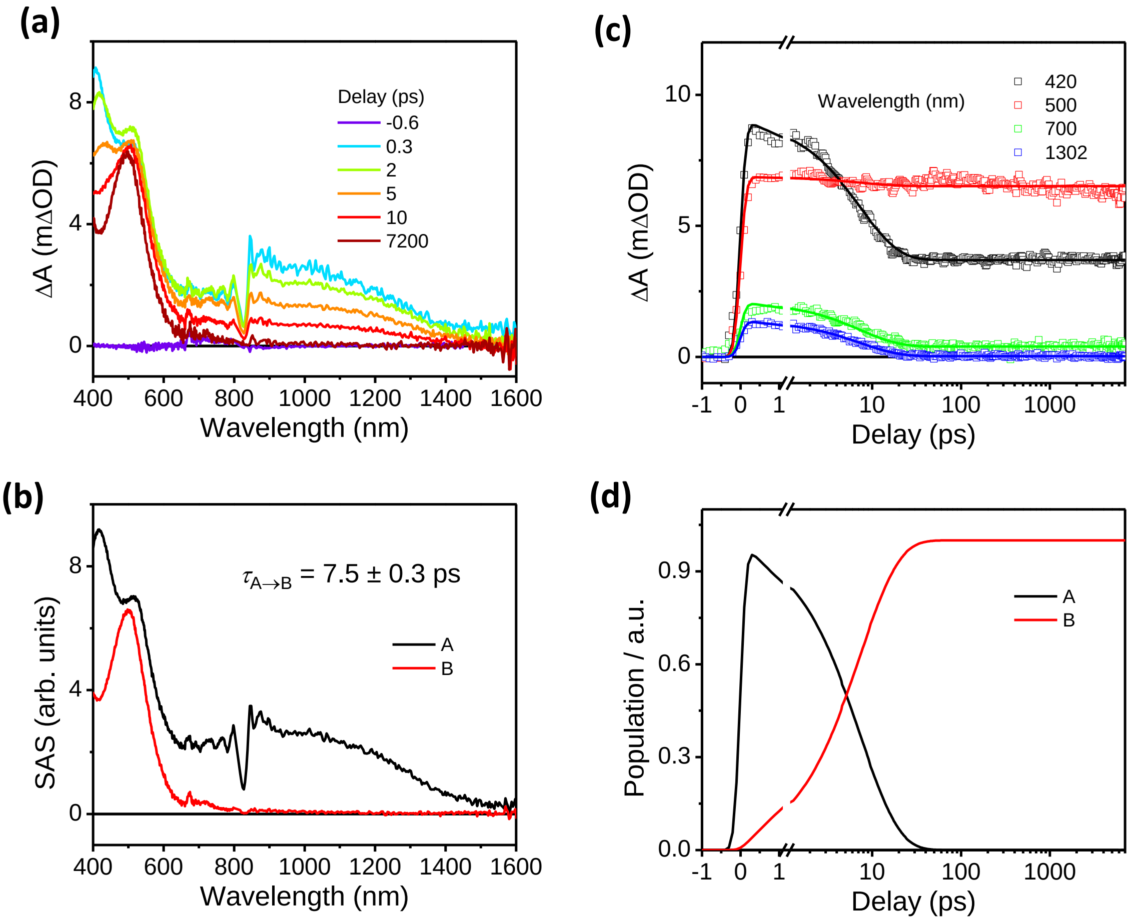

Figure S6. (a) fsTA spectra, (b) species-associated spectra (SAS), (c) multiple-wavelength fits, and (d) populations of kinetic states of **Br2NTE** in deaerated CH_2Cl_2 excited at 330 nm (A: S_1, B: T_1).

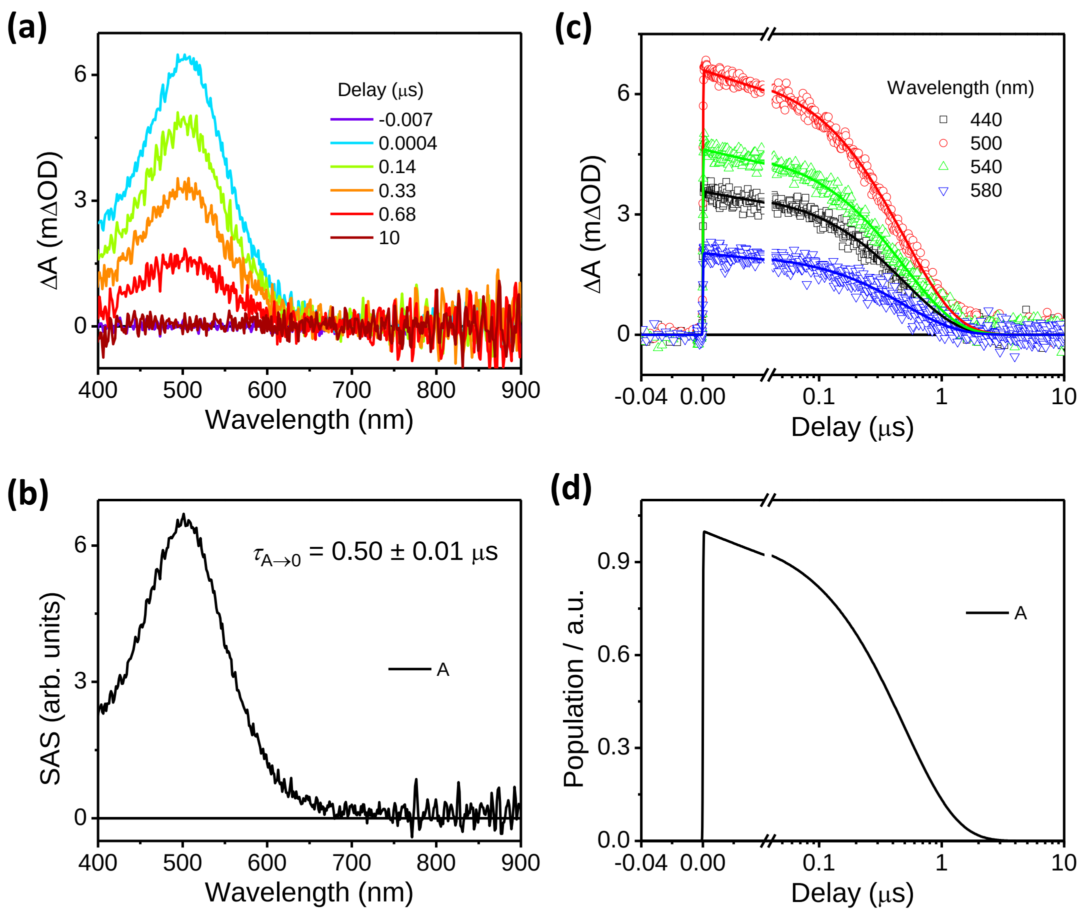

Figure S7. (a) nsTA spectra, (b) species-associated spectra (SAS), (c) multiple-wavelength fits, and (d) populations of kinetic states of **Br_2NTE** in deaerated CH_2Cl_2 excited at 330 nm (A: T_1).

- Transient absorption of **Br4NTE** in CH_2Cl_2:

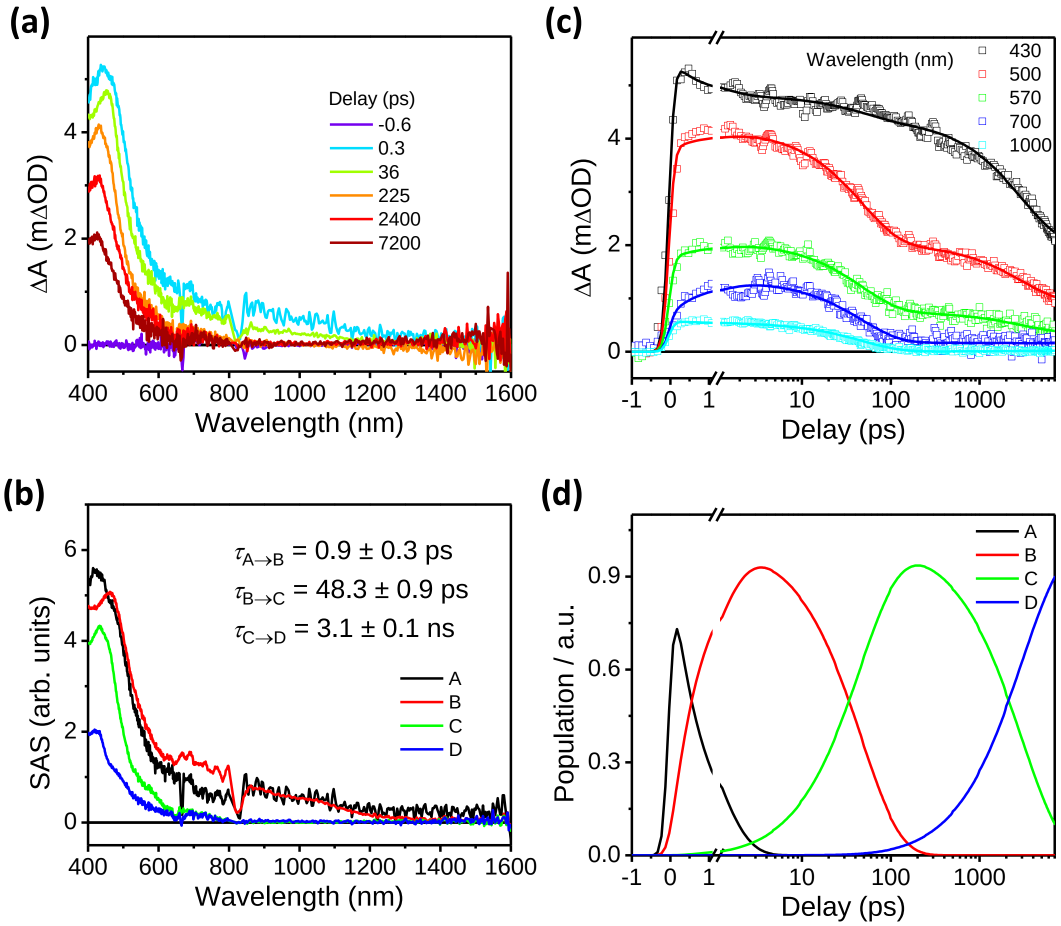

Figure S8. (a) fsTA spectra, (b) species-associated spectra (SAS), (c) multiple-wavelength fits, and (d) populations of kinetic states of **Br4NTE** in deaerated CH_2Cl_2 excited at 330 nm (A: hot S_1, B: relaxed S_1, C: hot T_1, D: relaxed T_1).

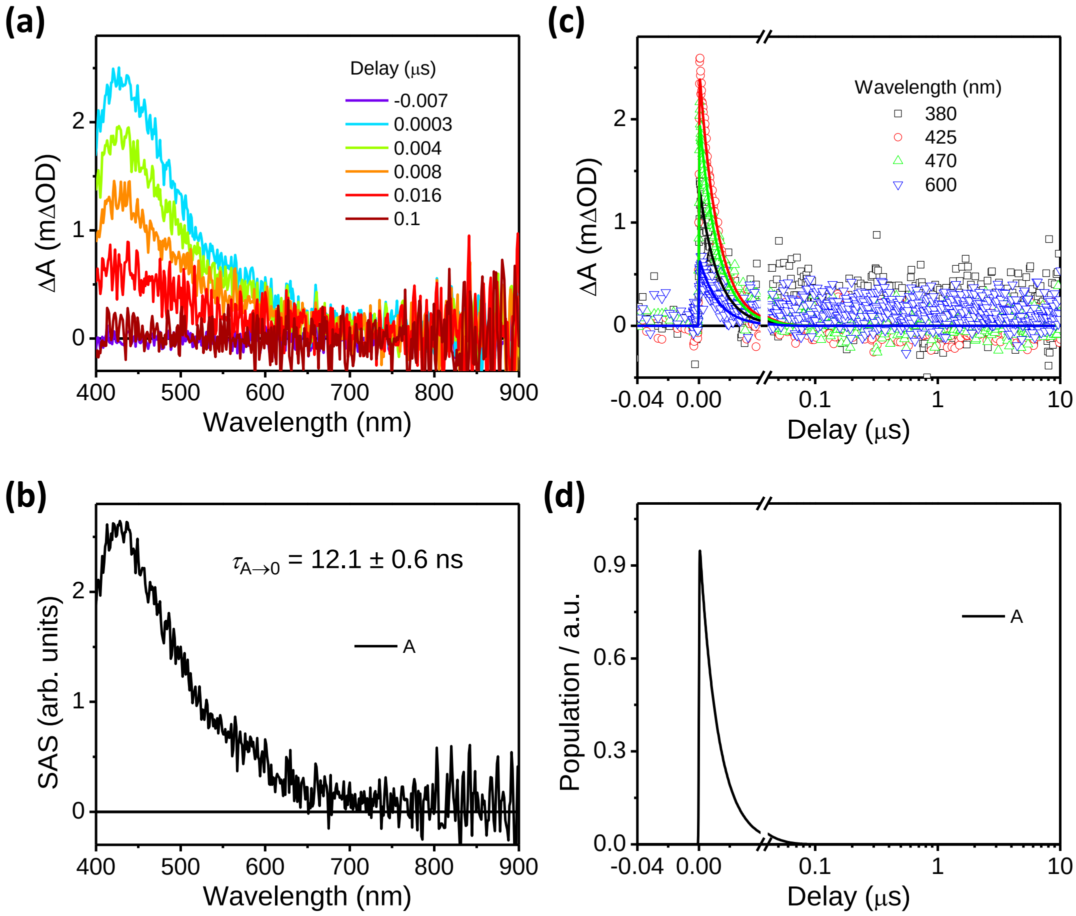

Figure S9. (a) nsTA spectra, (b) species-associated spectra (SAS), (c) multiple-wavelength fits, and (d) populations of kinetic states of **Br_4NTE** in deaerated CH_2Cl_2 excited at 330 nm (A: T_1).

6. Excitation spectra

Figure S10 shows the photoluminescence excitation spectra of the crystalline solids of **Br$_n$NTE**. The spectra were collected by monitoring the emission intensities at the phosphorescence λ_{max} for brominated samples (**Br$_n$NTE**, n = 1, 2, 4); for non-phosphorescent **Br$_0$NTE**, its weak fluorescence at 400 nm was used instead. The excitation spectrum provides information about the materials' electronic absorption responsible for the observed emission. The excitation bands of the solid samples are clearly red-shifted and broadened compared to the solution-phase spectra. Such a spectral change is common for chromophores transitioning from the gas/solution phase into the solid state. No significant difference between chromophores with or without halogen bonds was observed. In hindsight, the absence of such a difference is reasonable as the electronic transition is strongly π-π* in nature and centred on the naphthalene core (see also section 7) and the Br···O interactions are not particularly strong (as partially supported by the absence of phosphorescence from highly concentrated samples). Bowling and Anna and their co-workers similarly reported the weak influence of halogen bonding on the electronic absorption spectra for molecules displaying intramolecular halogen bonds (Bowling's case) and halogen bonds with solvents (Anna's case).[10, 11]

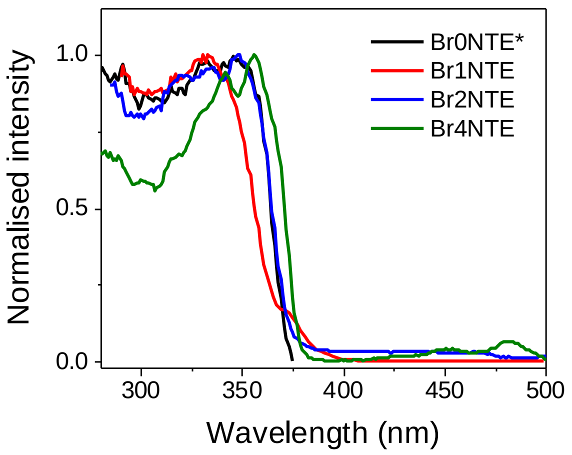

Figure S10. Photoluminescence excitation spectra of the crystalline solids of **Br$_n$NTE**. The spectra were collected by monitoring the phosphorescence intensities at the λ_{max} for **Br$_1$NTE**, **Br$_2$NTE**, and **Br$_4$NTE**; and the fluorescence intensities at 400 nm for **Br$_0$NTE**.

Additionally, we would like to point out that the non-zero intensity in the 400–500 nm region for **Br$_2$NTE** and **Br$_4$NTE** in Figure S10 is likely due to the direct $S_0 \rightarrow T_n$ absorption enabled by the heavy-atom effect. The observation of the formally forbidden $S_0 \rightarrow T_n$ transition in phosphorescence excitation spectra has been reported.[12, 13]

7. Computation details

The crystals of **Br$_n$NTE** (n = 0, 1, 2, 4) were optimised using periodic boundary conditions DFT as implemented in Quantum Espresso.[14] The PBE functional with dispersion correction (D2) was used with a plane-wave cut-off of 60 Ry. The Monkhorst-Pack k-point grids were chosen based on the unit cell shapes (2×1×2 for **Br$_0$NTE** and 1×1×1 for **Br$_1$NTE**, **Br$_2$NTE** and **Br$_4$NTE**). The molecules within unit cells were fully relaxed, whilst the unit cell dimensions were kept fixed at their experimental values.

Clusters of 97 **Br$_n$NTE** molecules (5238 atoms) were extracted from the optimised crystals. Singlet and triplet states of the **Br$_n$NTE** clusters were considered applying the ONIOM (QM:MM) method as implemented in Gaussian 16.[15] We have considered a two-layer representation with 1–3 central molecules and their closest intermolecular contacts treated at the ωB97X-D/6-31G(d) level of theory (QM part) and the rest of the cluster using the OPLS-AA force field (MM part)[16] (Figure S11). We used CM5 atomic charges[17] for the force-field derived from HF/6-31G(d) calculations. The QM region was completely relaxed while the MM part was kept in their crystal geometries. The S_0, S_1 states were optimised at the (TD-)ωB97X-D /6-31G(d) level of theory. In the case of T_1 states, the Tamm-Dancoff approximation (TDA) was applied to avoid problems with triplet instabilities. Single-point calculations were performed at the TDA-ωB97X-D/6-311+G(d,p) level of theory. The spin-orbit coupling matrix elements between the first five triplet states (T_1–T_5) and S_0 and S_1 states were computed at the S_1 and T_1 structures optimised at the QM/MM level. These calculations were based on Casida-type TDA-ωB97X-D/6-311+G(d,p) wave functions and the Breit-Pauli spin-orbit Hamiltonian, as implemented in the PySOC code.[18]

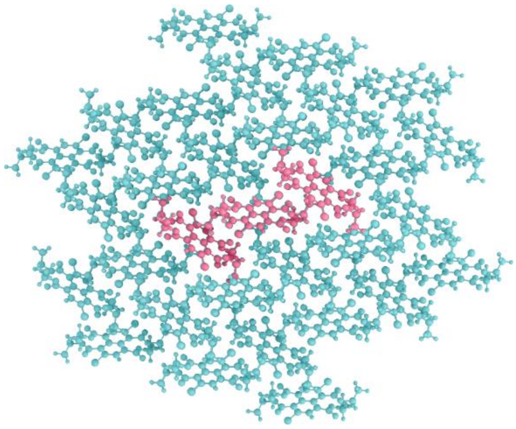

Figure S11. Illustration of the cluster models considered in the QM/MM calculations.

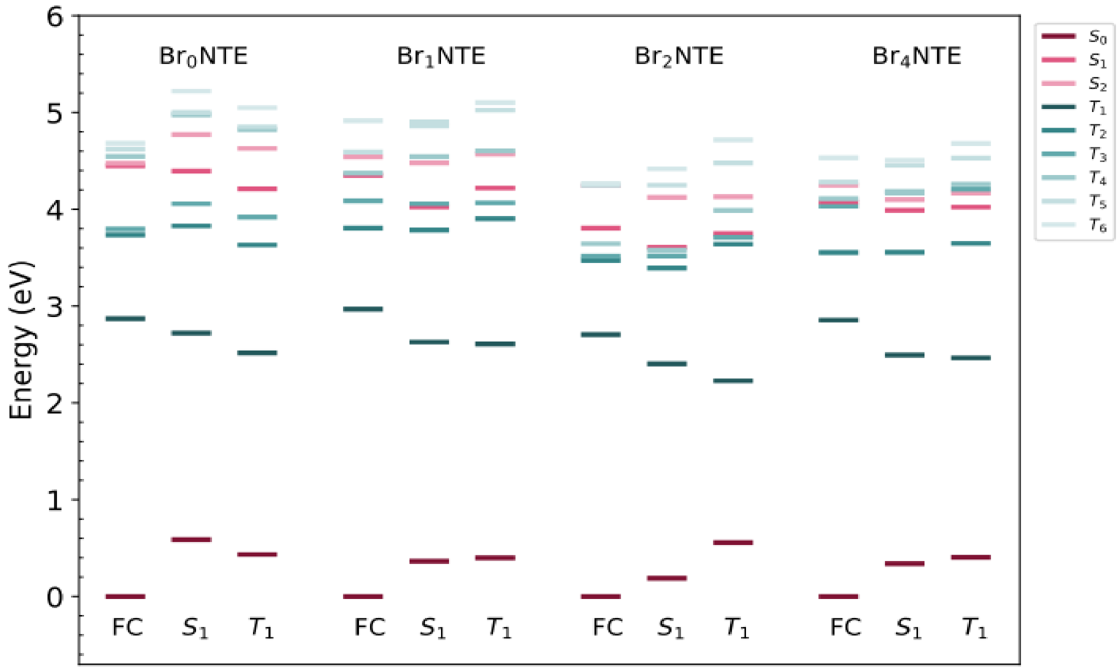

Figure S12. TDA-ωB97X-D/6-311+G(d,p)/OPLS-AA relative excitation energies (*E* in eV) at the S_0 (Franck Condon, FC), S_1 or T_1 geometries.

Table S4. TDA-ωB97X-D/6-311+G(d,p)/OPLS-AA excitation energies (*E* in eV) of the lowest-lying singlet and triplet states at the S_0 geometries of **Br$_n$NTE** molecules optimised at ωB97X-D/6-31G(d)/OPLS-AA level.

E (eV)	**Br$_0$NTE**	**Br$_1$NTE**	**Br$_2$NTE**	**Br$_4$NTE**
S_1	4.4482	4.3521	3.8048	4.0764
S_2	4.4776	4.5435	4.2449	4.2679
T_1	2.8729	2.9688	2.7975	2.8566
T_2	3.7368	3.8049	3.4749	3.5543
T_3	3.7956	4.0897	3.5167	4.0320
T_4	4.5474	4.3737	3.6453	4.1114
T_5	4.6199	4.5909	4.2466	4.2829
T_6	4.6849	4.9168	4.2677	4.5329

Table S5. TDA-ωB97X-D/6-311+G(d,p)/OPLS-AA excitation energies (*E* in eV) of the lowest-lying singlet and triplet states at the S_1 geometries of **Br_nNTE** molecules.

E (eV)	**Br_0NTE**	**Br_1NTE**	**Br_2NTE**	**Br_4NTE**
S_1	3.8071	3.6995	3.4190	3.6556
S_2	4.185	4.1173	3.9364	3.7681
T_1	2.1354	2.2653	2.2148	2.1625
T_2	3.2417	3.4207	3.2046	3.2223
T_3	3.4706	3.6926	3.3314	3.8408
T_4	4.3888	4.1803	3.3909	3.8497
T_5	4.4132	4.4953	4.0618	4.1204
T_6	4.6351	4.5400	4.2311	4.1727

Table S6. TDA-ωB97X-D/6-311+G(d,p)/OPLS-AA excitation energies (*E* in eV) of the lowest-lying singlet and triplet states at the T_1 geometries of **Br_nNTE** molecules optimised at TDA-ωB97X-D/6-31G(d)/OPLS-AA level.

E (eV)	**Br_0NTE**	**Br_1NTE**	**Br_2NTE**	**Br_4NTE**
S_1	3.776	3.7919	3.1916	3.6188
S_2	4.195	4.1728	3.5739	3.7634
T_1	2.0831	2.2091	1.6719	2.0614
T_2	3.1978	3.5067	3.0839	3.2438
T_3	3.4861	3.6689	3.1548	3.8032
T_4	4.388	4.204	3.4313	3.8572
T_5	4.4183	4.6268	3.9242	4.1258
T_6	4.6153	4.7055	4.1616	4.2775

Table S7. Spin-orbit couplings ($\langle S_i|H_{SO}|T_j\rangle$ in cm^{-1}) computed at the S_1 and T_1 geometries (in parentheses) of **Br$_n$NTE** monomers applying TDA-ωB97X-D/6-311+G(d,p) wave functions.

$\langle S_i\|H_{SO}\|T_j\rangle$	**Br$_0$NTE**	**Br$_1$NTE**	**Br$_2$NTE**	**Br$_4$NTE**
$\langle S_0\|H_{SO}\|T_1\rangle$	0.01	6.02 (3.22)	0.35 (142.27)	0.28 (0.38)
$\langle S_1\|H_{SO}\|T_1\rangle$	0.82	8.22 (7.05)	68.74 (51.51)	20.30 (19.82)
$\langle S_1\|H_{SO}\|T_2\rangle$	0.04	10.87 (7.45)	0.93 (94.90)	42.79 (43.08)
$\langle S_1\|H_{SO}\|T_3\rangle$	0.52	15.49 (9.88)	166.79 (361.27)	3.55 (0.11)
$\langle S_1\|H_{SO}\|T_4\rangle$	0.86	9.02 (7.39)	106.08 (139.78)	30.93 (33.55)
$\langle S_1\|H_{SO}\|T_5\rangle$	0.22	100.61 (85.22)	1.57 (217.08)	0.35 (0.33)

TD-DFT calculations of these chromophores in CH_2Cl_2 (PCM solvation model) or in the solid state (ONIOM) suggested that the absorption in the 300–350 nm region observed experimentally is contributed by two transitions ($S_0 \rightarrow S_1$ and $S_0 \rightarrow S_2$). Please note that these calculations were done at the Franck–Condon geometry and did not take the inter-chromophore dipolar interactions (for the solids) or vibrational effects (seems pronounced in **Br$_4$NTE**) into consideration. Since TD-DFT results reproduced the overall shape of the UV-Vis spectra and the relative intensities of the high-energy bands (<300 nm) and low-energy bands (>300 nm) (Figure S13; cf. Fig. 2a), we believe that the TD-DFT calculations can provide qualitative information regarding the nature of the transitions.

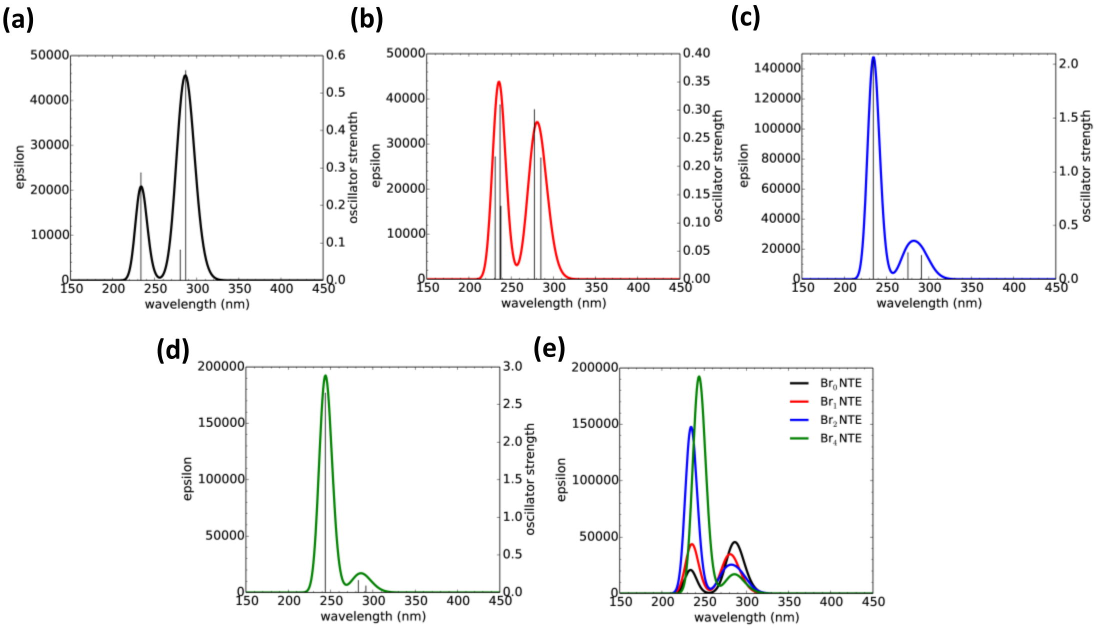

Figure S13. TD-DFT computed absorption spectra for (a) **Br$_0$NTE**, (b) **Br$_1$NTE**, (c) **Br$_2$NTE**, and (d) **Br$_4$NTE**. (e) The overlay of computed spectra.

In all cases, the transitions are dominantly π-π*, and these characters do not seem to be affected by halogen bonding (Table S8 and Figure S14). For **Br$_0$NTE**, there is a small n-π* (C=O) contribution in S_1, which has a much higher oscillator strength (*f*) compared to S_2. For brominated compounds, the oscillator strengths of these two states are more comparable, consistent with the solution-phase spectra where two bands were observed. For **Br$_1$NTE** and **Br$_2$NTE**, the transitions to the S_1 states have some contribution from Br, but less so for S_2. For **Br$_4$NTE**, the transitions to S_1 and S_2 states both involve Br-centred orbitals (to a different extent in solid or in solution, however).

Table S8. TD-DFT (ωB97X-D/6-311+G(d,p)) computed transition energy (eV) and oscillator strength (*f*) of **Br$_n$NTE**.

	State	**Br$_0$NTE**	**Br$_1$NTE**	**Br$_2$NTE**	**Br$_4$NTE**
Solution	S_1	4.32 (*f*=0.56)	4.35 (*f*=0.21)	4.26 (*f*=0.23)	4.25 (*f*=0.09)
	S_2	4.42 (*f*=0.08)	4.47 (*f*=0.30)	4.50 (*f*=0.25)	4.39 (*f*=0.16)
Crystal	S_1	4.45 (*f*=0.31)	4.35 (*f*=0.10)	3.80 (*f*=0.11)	4.08 (*f*=0.02)
	S_2	4.48 (*f*=0.09)	4.54 (*f*=0.17)	4.24 (*f*=0.11)	4.27 (*f*=0.04)

(a) Solution (CH_2Cl_2)

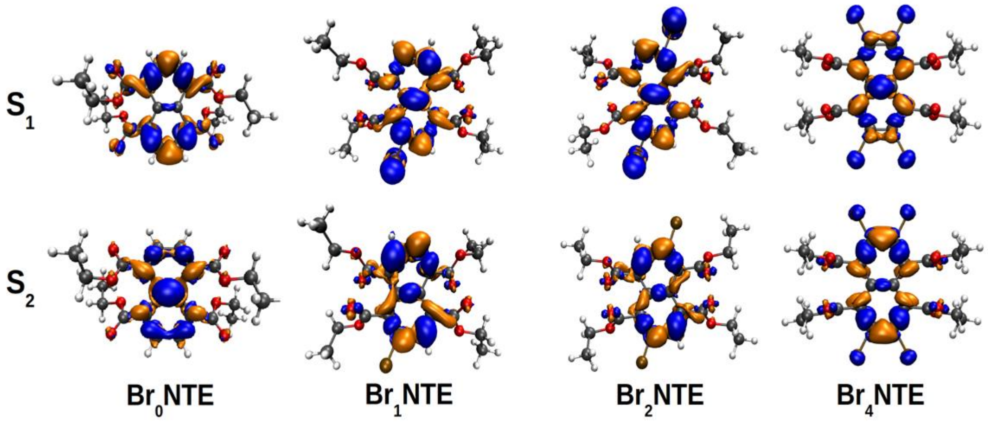

(b) Solid (ONIOM)

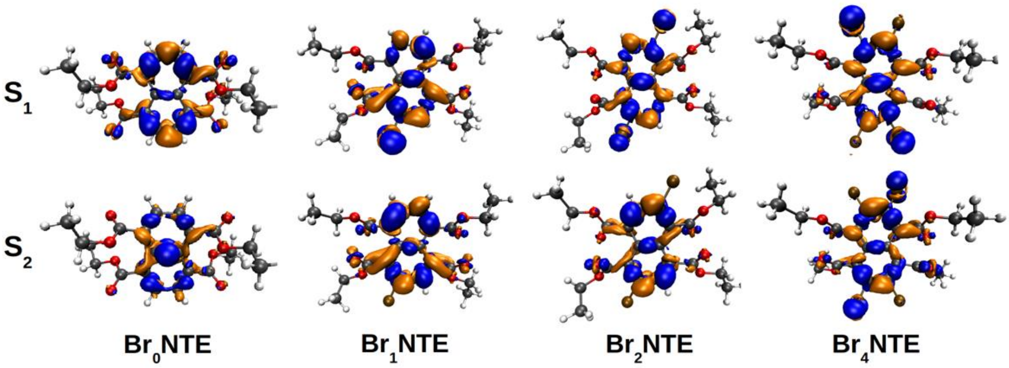

Figure S14. Electron density difference plots (0.001 e Bohr^{-3} isovalue) between the selected excited states (S_1 or S_2) and the ground state at the Franck–Condon geometry.

Excitation into the higher excited states (S_n, $n \geq 2$) is expected to be followed by rapid internal conversion to form the S_1 (Kasha's rule). As S_1 either has some n-π* (C=O) (**Br_0NTE**) or bromo (**Br_1NTE**, **Br_2NTE**, or **Br_4NTE**) contributions, intersystem crossing can take place effectively even if the system was initially prepared at the higher excited states. This is consistent with the excitation spectra discussed in section 6 (Figure S10), where there is no apparent energy cut off to indicate the nonradiative character of the higher excited states.

8. AIM analysis of C–Br···OEt and C–Br···O=C interactions

Chromophore rigidity contributes partly to the RTP process. Stronger halogen bonding should impose a stronger structural constraint on the chromophores. As the bonding strength is not directly measurable, we used the AIM ("atoms in molecules") analysis, performed at ωB97X-D/6-311+G(d,p) level of theory, to investigate the electron density characteristics at the bond critical points (ρ_{BCP}, see Table S9; based on the DFT optimised structures).[19, 20] It has been shown that higher ρ_{BCP} correlates with stronger non-covalent interactions. Across the board, higher ρ_{BCP} was found for C–Br···O=C, which is in line (intuitively) with the shorter *d*(Br···O) distance. The positive $\nabla^2\rho_{BCP}$ values indicate a depletion of the density, typical of weak interactions. This analysis suggests that C–Br···O=C may contribute more to the RTP process.

Table S9. Electron densities at the bond critical points (ρ_{BCP} in 10^{-2} a.u.) and their Laplacians ($\nabla^2\rho_{BCP}$ in 10^{-2} a.u.) obtained with AIM theory.

	Bond	ρ_{BCP}	$\nabla^2\rho_{BCP}$	*d*(Br···O) / Å
**Br$_1$NTE**	C–Br···O=C	0.663	2.552	3.29
	C–Br···O–C_2H_5	0.662	2.459	3.32
**Br$_2$NTE**	C–Br···O=C	0.741	2.851	3.23
	C–Br···O–C_2H_5	0.717	2.643	3.29
**Br$_4$NTE**	C–Br···O=C	1.130	4.032	3.04
	C–Br···O–C_2H_5	0.244	0.959	3.86

9. Temperature-dependent phosphorescence

A preliminary test on the temperature dependence indicated that the RTP intensity of **Br_2NTE** is diminished at high temperatures (however, not much changes were visually observable at 30–50 ºC) and restored at room temperature (Figure S15). This is a reversible process and can be repeated several times.

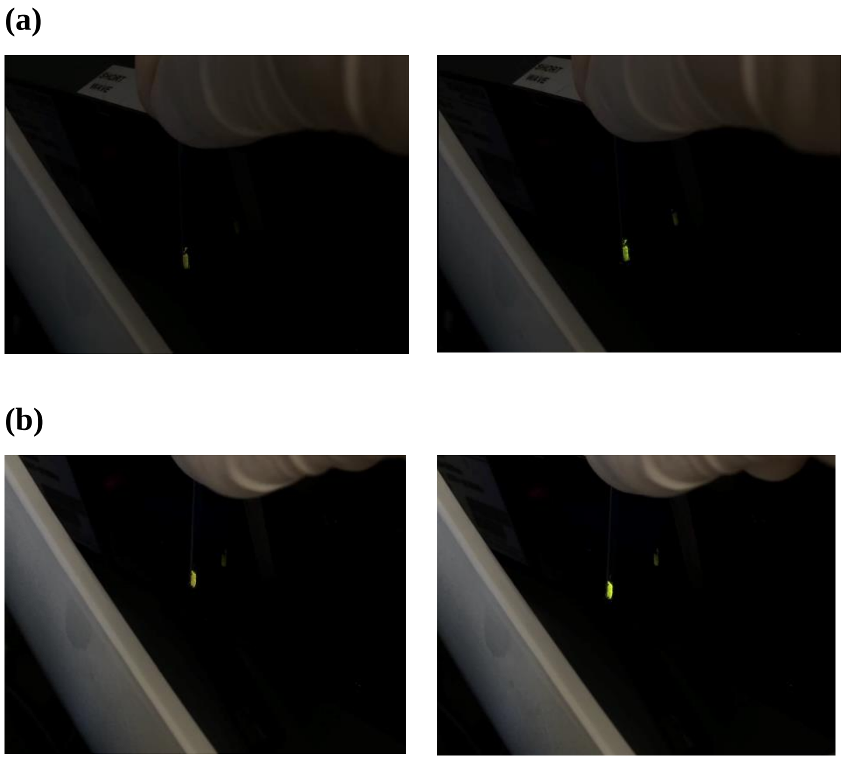

Figure S15. Photographs of crystalline **Br_2NTE** under 365 nm irradiation at high (left column, close to 100 ºC) and low (right column, close to 30 ºC) temperatures. Panels (a) and (b) represent two heating–cooling cycles.

The temperature dependence reflects the non-covalent and dynamic nature of halogen bonds in constraining the excited-state geometry. Moreover, this observation suggested that these materials can be mixed with a fluorescent emitter (with weak temperature dependence) to display photoluminescence of different colours at variable temperatures. This thermochromic behaviour can be used to code security information that is only visible after heating. However, a proper illustration of such an application and the analysis of the corresponding structural and luminescence changes would require a suitable instrument to offer accurate temperature control of the solids.

10. ^{1}H and ^{13}C NMR Spectra

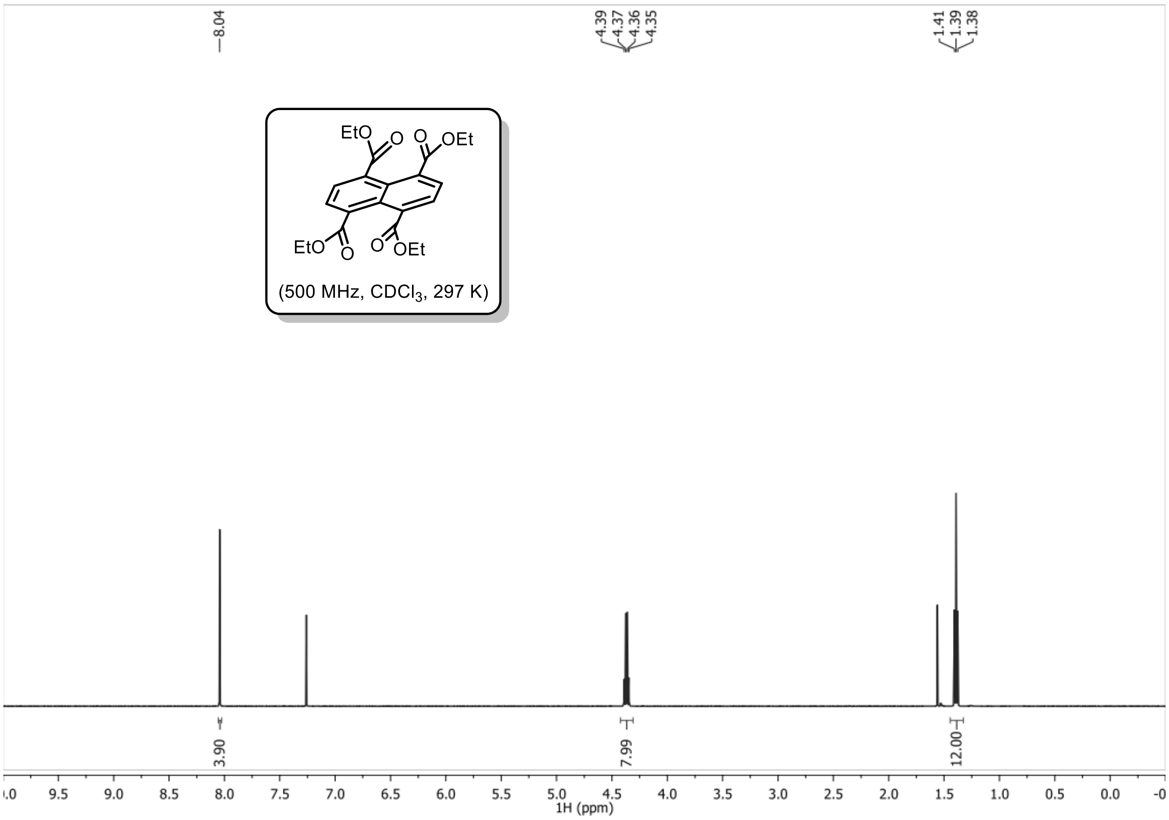

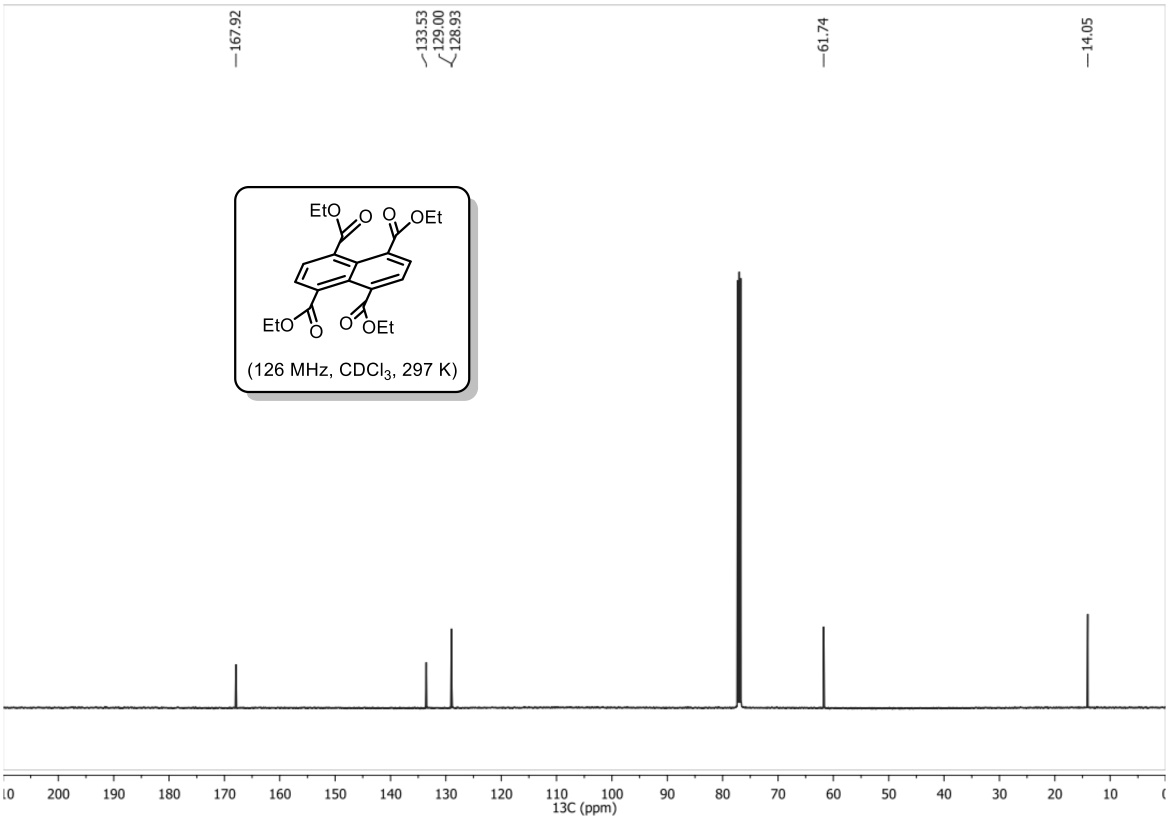

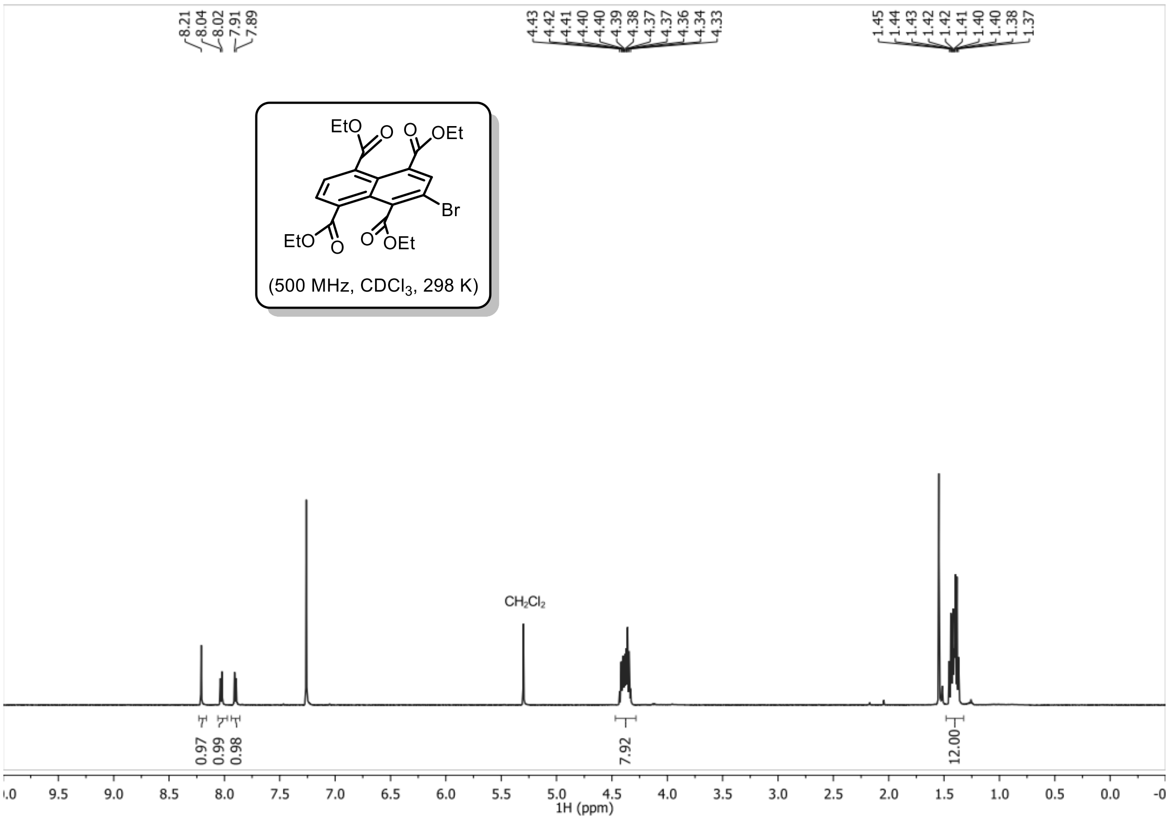
8.21
8.04
8.02
7.91
7.89
4.43
4.42
4.41
4.40
4.40
4.39
4.38
4.37
4.37
4.36
4.34
4.33
1.45
1.44
1.43
1.42
1.42
1.41
1.40
1.40
1.38
1.37
EtO
O
O
OEt
Br
EtO
O
O
OEt
(500 MHz, CDCl3, 298 K)
CH2Cl2
0.97
0.99
0.98
7.92
12.00
1H (ppm)

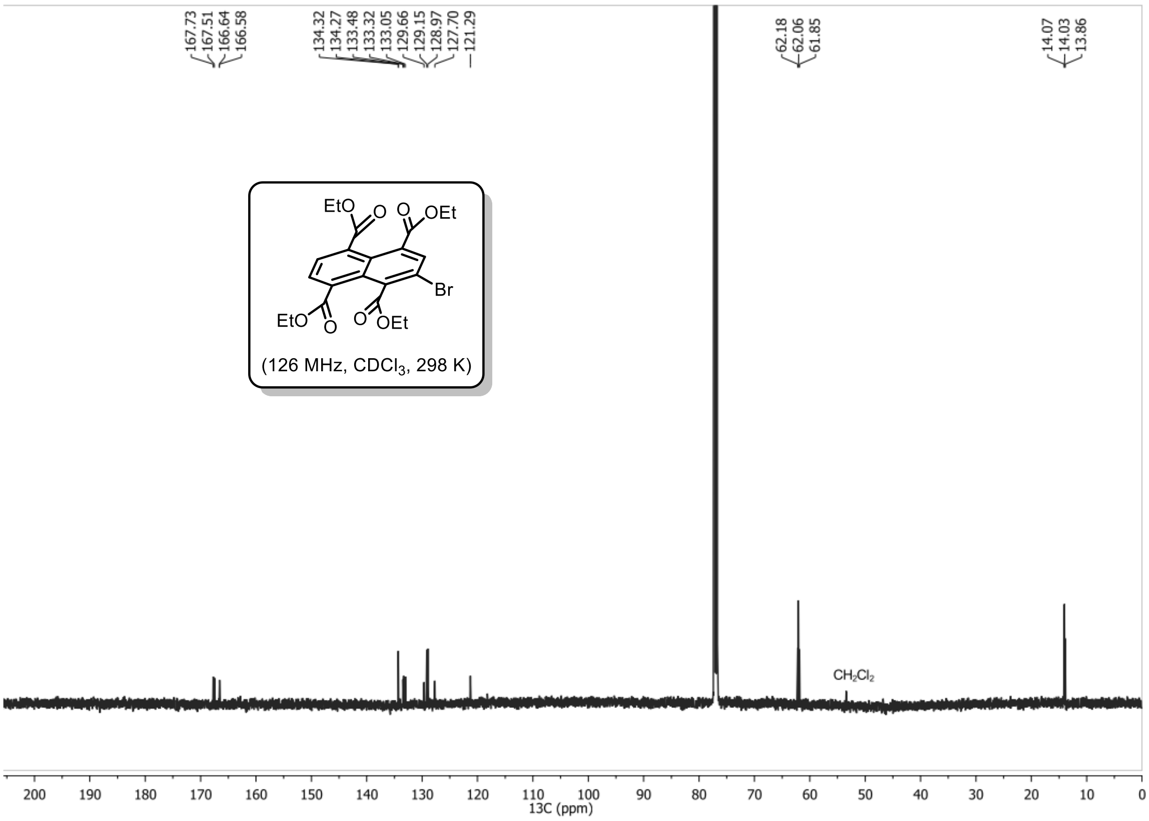
167.73
167.51
166.64
166.58
134.32
134.27
133.48
133.32
133.05
129.66
129.15
128.97
127.70
121.29
62.18
62.06
61.85
14.07
14.03
13.86
EtO
O
O
OEt
Br
EtO
O
O
OEt
(126 MHz, CDCl3, 298 K)
CH2Cl2
13C (ppm)

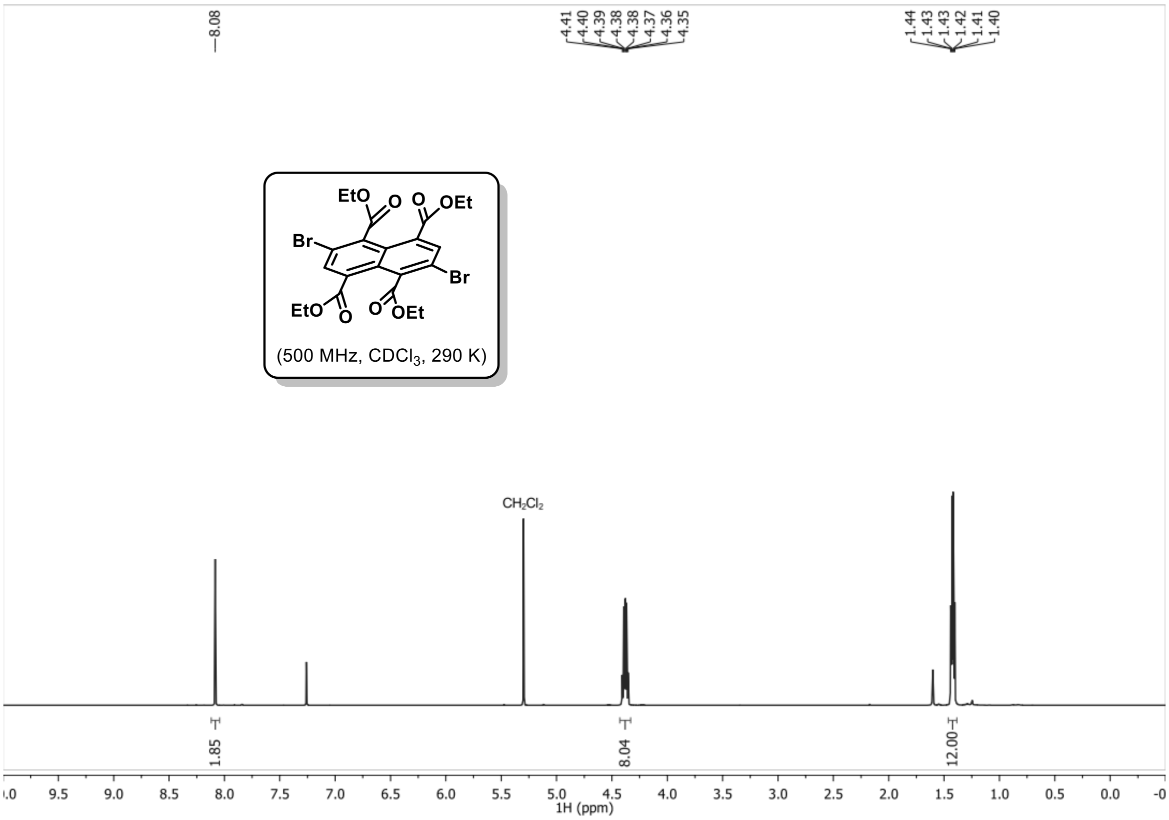
8.08
4.41 4.40 4.39 4.38 4.38 4.37 4.36 4.35
1.44 1.43 1.43 1.42 1.41 1.40
EtO O O OEt
Br
Br
EtO O O OEt
(500 MHz, $CDCl_3$, 290 K)
CH_2Cl_2
1.85
8.04
12.00
1H (ppm)

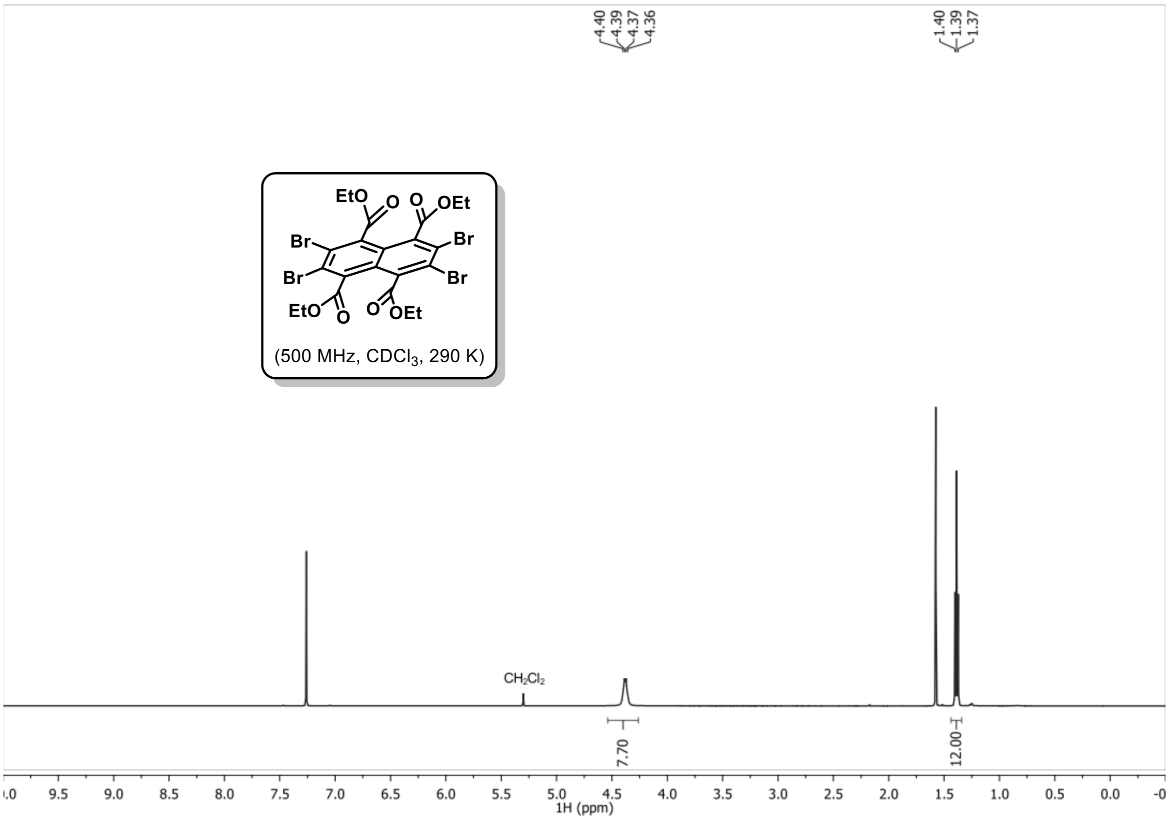
4.40 4.39 4.37 4.36
1.40 1.39 1.37
EtO O O OEt
Br Br
Br Br
EtO O O OEt
(500 MHz, $CDCl_3$, 290 K)
CH_2Cl_2
7.70
12.00
1H (ppm)

11. References

1. G. M. Sheldrick, *Acta Crystallogr., Sect. A: Found. Crystallogr.*, 2015, **71**, 3-8.
2. G. M. Sheldrick, *Acta Crystallogr., Sect. C: Struct. Chem.*, 2015, **71**, 3-8.
3. O. V. Dolomanov, L. J. Bourhis, R. J. Gildea, J. A. K. Howard and H. Puschmann, *J. Appl. Crystallogr.*, 2009, **42**, 339-341.
4. R. M. Young, S. M. Dyar, J. C. Barnes, M. Juricek, J. F. Stoddart, D. T. Co and M. R. Wasielewski, *J. Phys. Chem. A*, 2013, **117**, 12438-12448.
5. R. S. K. Kishore, V. Ravikumar, G. Bernardinelli, N. Sakai and S. Matile, *J. Org. Chem.*, 2008, **73**, 738-740.
6. M. Sasikumar, Y. V. Suseela and T. Govindaraju, *Asian J. Org. Chem.*, 2013, **2**, 779-785.
7. C. Roger and F. Wurthner, *J. Org. Chem.*, 2007, **72**, 8070-8075.
8. Y. Ma, X. Zhang, S. Stappert, Z. Yuan, C. Li and K. Mullen, *Chem. Commun.*, 2017, **53**, 5310-5313.
9. A. A. Berezin, A. Sciutto, N. Demitri and D. Bonifazi, *Org. Lett.*, 2015, **17**, 1870-1873.
10. Y. Lee, R. M. Malamakal, D. M. Chenoweth and J. M. Anna, *J. Phys. Chem. Lett.*, 2020, **11**, 877-884.
11. D. L. Widner, E. R. Robinson, A. B. Perez, H. G. Vang, R. A. Thorson, Z. L. Driscoll, S. M. Giebel, C. W. Berndt, E. Bosch, E. D. Speetzen and N. P. Bowling, *Eur. J. Org. Chem.*, 2017, **2017**, 5739-5749.
12. J. L. Tomer, K. W. Holtzclaw, D. W. Pratt and L. H. Spangler, *J. Chem. Phys.*, 1988, **88**, 1528-1538.
13. L. M. Hoffelt, M. G. Springer and S. Drucker, *J. Chem. Phys.*, 2008, **128**, 104312.
14. P. Giannozzi, S. Baroni, N. Bonini, M. Calandra, R. Car, C. Cavazzoni, D. Ceresoli, G. L. Chiarotti, M. Cococcioni, I. Dabo, A. Dal Corso, S. de Gironcoli, S. Fabris, G. Fratesi, R. Gebauer, U. Gerstmann, C. Gougoussis, A. Kokalj, M. Lazzeri, L. Martin-Samos, N. Marzari, F. Mauri, R. Mazzarello, S. Paolini, A. Pasquarello, L. Paulatto, C. Sbraccia, S. Scandolo, G. Sclauzero, A. P. Seitsonen, A. Smogunov, P. Umari and R. M. Wentzcovitch, *J. Phys.: Condens. Matter.*, 2009, **21**, 395502.
15. M. J. Frisch, G. W. Trucks, H. B. Schlegel, G. E. Scuseria, M. A. Robb, J. R. Cheeseman, G. Scalmani, V. Barone, G. A. Petersson, H. Nakatsuji, X. Li, M. Caricato, A. V. Marenich, J. Bloino, B. G. Janesko, R. Gomperts, B. Mennucci, H. P. Hratchian, J. V. Ortiz, A. F. Izmaylov, J. L. Sonnenberg, Williams, F. Ding, F. Lipparini, F. Egidi,

J. Goings, B. Peng, A. Petrone, T. Henderson, D. Ranasinghe, V. G. Zakrzewski, J. Gao, N. Rega, G. Zheng, W. Liang, M. Hada, M. Ehara, K. Toyota, R. Fukuda, J. Hasegawa, M. Ishida, T. Nakajima, Y. Honda, O. Kitao, H. Nakai, T. Vreven, K. Throssell, J. A. Montgomery Jr., J. E. Peralta, F. Ogliaro, M. J. Bearpark, J. J. Heyd, E. N. Brothers, K. N. Kudin, V. N. Staroverov, T. A. Keith, R. Kobayashi, J. Normand, K. Raghavachari, A. P. Rendell, J. C. Burant, S. S. Iyengar, J. Tomasi, M. Cossi, J. M. Millam, M. Klene, C. Adamo, R. Cammi, J. W. Ochterski, R. L. Martin, K. Morokuma, O. Farkas, J. B. Foresman and D. J. Fox, Gaussian 16 Rev. C.01, Ver. Gaussian Inc., Wallingford, CT, 2016. available at

16. W. L. Jorgensen, D. S. Maxwell and J. TiradoRives, *J. Am. Chem. Soc.*, 1996, **118**, 11225-11236.
17. A. V. Marenich, S. V. Jerome, C. J. Cramer and D. G. Truhlar, *J. Chem. Theory Comput.*, 2012, **8**, 527-541.
18. X. Gao, S. M. Bai, D. Fazzi, T. Niehaus, M. Barbatti and W. Thiel, *J. Chem. Theory Comput.*, 2017, **13**, 515-524.
19. T. Lu, Multiwfn -- A Multifunctional Wavefunction Analyser, Ver. 3.3.8, 2015. available at http://multiwfn.codeplex.com
20. T. Lu and F. Chen, *J. Comput. Chem.*, 2012, **33**, 580-592.
